# On the Prediction of the Flow Behavior of Metals and Alloys at a Wide Range of Temperatures and Strain Rates Using Johnson–Cook and Modified Johnson–Cook-Based Models: A Review

**DOI:** 10.3390/ma16041574

**Published:** 2023-02-13

**Authors:** Abdallah Shokry, Samer Gowid, Hasan Mulki, Ghais Kharmanda

**Affiliations:** 1 Department of Mechanical Engineering, Faculty of Engineering, Fayoum University, Fayoum 63514, Egypt; 2 Department of Mechanical and Industrial Engineering, Qatar University, Doha 2713, Qatar; 3 College of Engineering and Technology, American University of the Middle East, Egaila 54200, Kuwait; 4 Mechanics Laboratory of Normandy, INSA Rouen Normandie, 76800 Saint Etienne du Rouvray, France; 5 3D Printing 4U (UG), 51103 Cologne, Germany

**Keywords:** hot deformation, elevated temperature, high strain rate, Johnson–Cook, modified Johnson–Cook, constitutive modeling

## Abstract

This paper reviews the flow behavior and mathematical modeling of various metals and alloys at a wide range of temperatures and strain rates. Furthermore, it discusses the effects of strain rate and temperature on flow behavior. Johnson–Cook is a strong phenomenological model that has been used extensively for predictions of the flow behaviors of metals and alloys. It has been implemented in finite element software packages to optimize strain, strain rate, and temperature as well as to simulate real behaviors in severe conditions. Thus, this work will discuss and critically review the well-proven Johnson–Cook and modified Johnson–Cook-based models. The latest model modifications, along with their strengths and limitations, are introduced and compared. The coupling effect between flow parameters is also presented and discussed. The various methods and techniques used for the determination of model constants are highlighted and discussed. Finally, future research directions for the mathematical modeling of flow behavior are provided.

## 1. Introduction

Hot deformation is one of the most well-known ways to enhance the mechanical properties of metals and alloys [1,2,3,4,5,6] via grain refinements [7,8,9,10], in which temperature is raised above recrystallization temperature during plastic deformation. The enhancement to the mechanical properties is controlled by work hardening, dynamic recovery (DRV), and dynamic recrystallization (DRX), which have a huge effect on the microstructure as well as the flow stress behavior of the alloys [11,12,13,14,15]. Accordingly, proper hot working parameters such as strain, strain rate, and temperature must be carefully chosen to achieve the desired mechanical properties [16,17,18,19,20]. The hot working parameters can be optimized by employing finite element simulations of the hot deformation process [21,22,23,24,25,26], in which the constitutive modeling of the flow stress behavior of alloys plays a significant role [27,28,29]. In addition to the importance of hot deformation, modeling the flow stress and plastic deformation of metals and alloys that are employed in applications under severe conditions such as very high strain rates (dynamic loadings) and different temperatures is also essential [30,31,32].

The constitutive models for the prediction of flow stress behavior at different strain rates and temperatures can be categorized as physical-based models [33,34,35,36], in which the physical aspects of material behavior are taken into consideration; phenomenological-based models [37,38,39,40,41], where empirical observations with mathematical functions establish the flow stress; and intelligence-based models [42,43,44,45,46], in which machine learning is used in the prediction of flow stress. In 1983, Johnson and Cook (JC) presented their well-known phenomenological constitutive model for the prediction of the flow behavior of materials at elevated temperatures and different strain rates [47]. The JC model contains three independent terms: strain hardening, strain rate, and thermal softening. One of the advantages of the JC model is that it has only five constants. Furthermore, it has been implemented in finite element simulation software packages for the prediction of flow stress in severe conditions such as elevated temperatures and high strain rates, as well as to optimize hot working parameters during hot deformation. The JC model has been commonly used for different materials such as nickel-based [48,49], iron-based [30,50], aluminum-based [51,52,53], magnesium-based [54,55,56], and titanium-based [57,58,59,60] alloys.

The high complexity of the non-linear behavior of flow stress at elevated temperatures and different strain rates for some alloys causes the JC model to fail to reach precise predictions from time to time [61,62,63]. The inaccurate predictions may be due to the fact that the JC model implements the three effects of hardening, strain rate, and thermal softening without any interaction between the three of them. Indeed, the strain, strain rate, and temperature are interconnected [64,65,66]. In order to improve the accuracy of predictions made by the original JC model for different materials, many modified JC-based models have been presented [48,53,56,63,67,68,69,70,71,72,73,74,75,76,77,78,79,80,81,82,83,84,85,86,87,88,89,90,91,92,93,94,95,96,97]. Therefore, this study will be limited to studying the predictability of JC and modified JC-based models.

In this article, the effect of strain rate and temperature on the flow stress behavior of metals and alloys is outlined. Subsequently, the constitutive modeling and accuracy of predictions at a wide range of temperatures and strain rates using original JC and over thirty modified JC-based models are critically reviewed and presented. Furthermore, the implemented methods and approaches that are used to determine the constants that constitute the models are explained. Finally, a summary based on the three terms of the original JC and modified JC-based models is presented. The future potential of this research area is also considered.

## 2. Flow Stress Behavior

**Figure 1:**
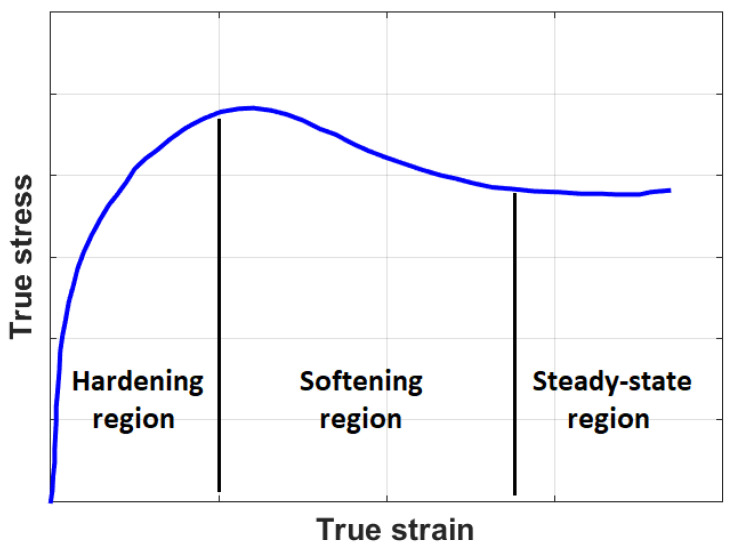
Common flow stress curve under hot deformation.

A common flow stress curve under hot deformation is shown in Figure 1. The figure shows that the flow stress behavior under hot deformation includes three regions: (i) the hardening region, in which a rapid increase in the flow stress up to the peak can be observed due to work hardening and DRV; (ii) the softening region, in which stress sharply falls due to the combined effect of DRV and DRX; and (iii) the steady-state region, in which steady stress overcomes as a result of the equilibrium between hardening and softening [16,98].

**Figure 2:**
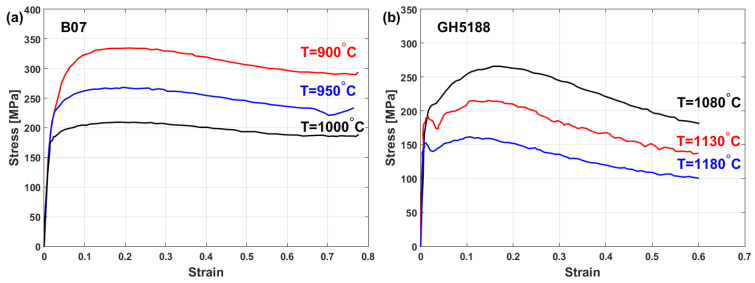
Flow stress curves during hot deformation at 0.1 s^−1^ for (**a**) B07 superalloy and (**b**) GH5188 superalloy.

The flow behaviors of the B07 [99] and GH5188 [100] superalloys at elevated temperatures and a strain rate of 0.1 s^−1^ are shown in Figure 2a and Figure 2b, respectively. As can be seen, the flow stress is extremely sensitive to temperature and passes the three regions that are defined in Figure 1. The obtained stress–strain curves for the B07 and GH5188 superalloys under hot deformation show that both alloys are greatly affected by hot working parameters, i.e., strain, strain rate, and temperature, in which stress decreases as the temperature increases and the strain rate decreases. Similarly, the same flow stress behavior under hot deformation has been reported for many different alloys, such as iron [101,102,103], titanium [104,105,106], aluminum [107,108,109,110], copper [111,112], nickel [113,114,115], and magnesium [116,117] alloys.

**Figure 3:**
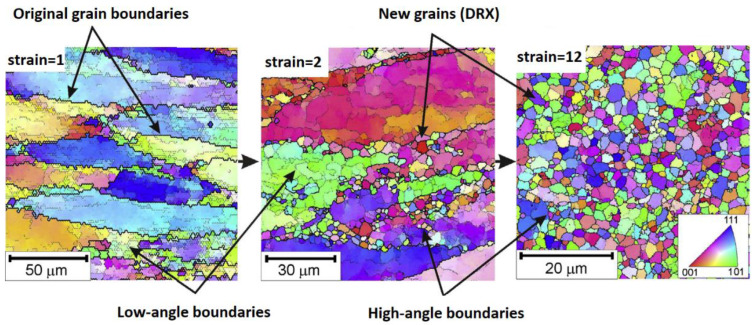
Evolution of new grains in AA1421 aluminum alloy due to DRX [123].

As a result of DRX, dislocation is formed due to the release of stored elastic energy in the hardening region, which results in homogeneous sub-grains. As the strain increases, the misorientations between sub-grains increase, and the sub-grains are turned into fine grains [118,119,120,121,122]. Figure 3 shows the formation of new fine grains in AA1421 aluminum alloy due to DRX, in which aluminum alloy was processed using equal channel angular extrusion (ECAP) repeatedly to a strain of 12 at 673 K.

### 2.1. Effect of Strain Rate

**Figure 4:**
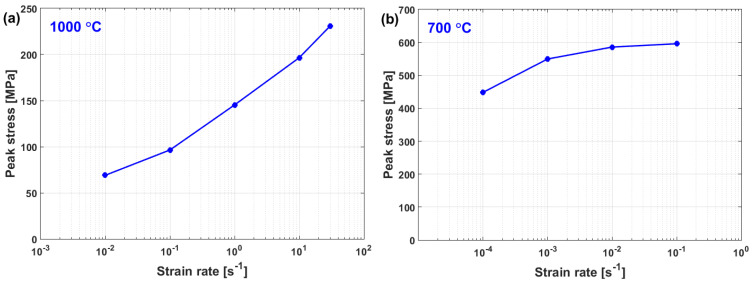
Peak stress vs. strain rate during hot deformation for (**a**) Fe-26Mn-6.2Al-0.05C steel and (**b**) Duplex cast steels.

Figure 4 shows the peak stress vs. strain rate for Fe-26Mn-6.2Al-0.05C steel alloy at 1000 °C [124] (cf. Figure 4a) and Duplex cast steel alloy at 700 °C [125] (cf. Figure 4b). The figure shows that, as the strain rate increases, the peak stress increases for both alloys. Similar findings can be found in [126,127,128,129,130]. As a matter of fact, this increase is attributed to the speedy rate of strain hardening, especially in the initial stage. Thus, the generation and multiplication of dislocations are accompanied by higher strain rates, in which high stresses are required due to dislocation interactions [131,132,133]. On the contrary, there is not enough time for DRV and DRX as restoration mechanisms [134,135].

**Figure 5:**
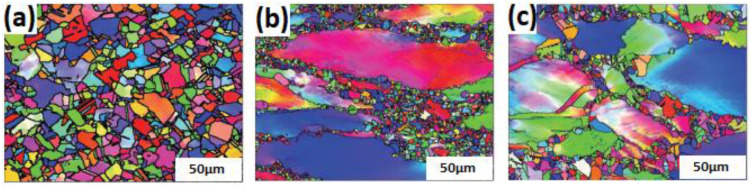
Inverse pole figure maps of microstructures using the EBSD technique on GH690 superalloy at 1000 °C and a true strain of 0.7 for (**a**) 0.001 s^−1^, (**b**) 0.1 s^−1^, and (**c**) 5 s^−1^ [136].

Shi et al. [136] studied the effect of strain rate on the hot deformation properties of the GH690 superalloy. Figure 5 shows that, with a lower strain rate (0.001 s^−1^), fine grains due to full DRX are developed. It also shows the formation of a few column grains in addition to the equiaxed grains as the strain rate increases (0.1 s^−1^ and 5 s^−1^). The effect of strain rate on the hot deformation of other alloys has been reported with the same findings, such as magnesium [137], CoCrFeMnNi high-entropy [138], nickel [139,140], aluminum [141], iron [142], and titanium [143] alloys.

### 2.2. Effect of Temperature

**Figure 6:**
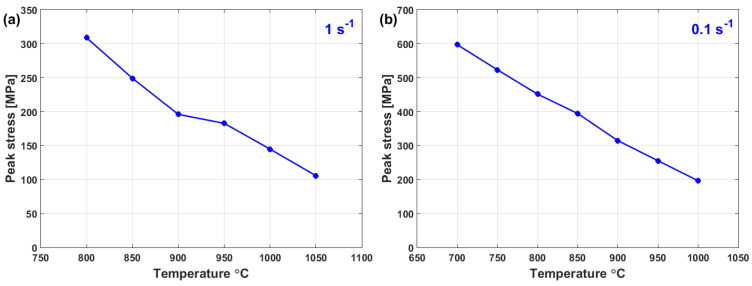
Peak stress vs. temperature during hot deformation for (**a**) Fe-26Mn-6.2Al-0.05C steel and (**b**) Duplex cast steel.

Figure 6 shows peak stress vs. temperature for Fe-26Mn-6.2Al-0.05C steel alloy [124] (Figure 6a) and Duplex cast steel alloy [125] (Figure 6b) at 1 s^−1^ and 0.1 s^−1^, respectively. As can be seen, the peak stress decreases as the temperature increases. Similar findings can be found in [144,145,146,147,148]. At high temperatures and low strain rates, the flow stress decreases due to the slow rate of both DRV and DRX [149,150], in which the interaction and annihilation of the dislocations can be guided by adequate thermal energy in plenty of time [151,152].

**Figure 7:**
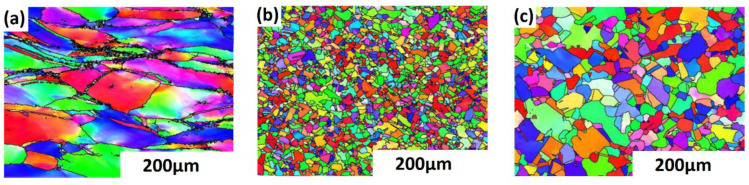
Inverse pole figure maps of microstructures of new nickel-based superalloy at 0.01 s^−1^ for (**a**) 950 °C, (**b**) 1050 °C, and (**c**) 1150 °C [153].

Song et al. [153] studied the effect of hot deformation and DRX on a new Ni-Cr-Co nickel-based superalloy. Figure 7 shows that increasing the temperature leads to the formation of fine grains. Elongated grains can be seen in Figure 7a, passing with DRX at grain boundaries in Figure 7b and, finally, complete DRX and the growth of fine grains, as in Figure 7c. The effect of temperature on different alloys has been noticed with the same findings, such as nickel [154,155], Al-Cu-Mg [156], titanium [157] Ni-Co [158], and aluminum [159] alloys.

## 3. Constitutive Models

In this section, the well-known JC and modified JC-based models are presented and carefully reviewed. In addition, the associated methods to determine the models’ constants are also considered.

### 3.1. Original Johnson–Cook Model

Johnson and Cook [47] introduced their famous model to predict the flow behavior of materials at high temperatures and different strain rates. The model is very simple, and three independent parts were presented. The three parts are (i) strain hardening, (ii) strain rate, and (iii) thermal softening. Only five constants are introduced to constitute the JC model. The model can be expressed as

(1) $$\sigma =\left(A+B\varepsilon^{n}\right)\left(1+C\ln\varepsilon^{\cdot *}\right)\left(1-T^{*}{}^{m}\right)$$

where σ is the flow stress, and ε is the plastic strain. The strain-hardening part is introduced by the term $A+B\varepsilon^{n}$, where A is yield stress, and B and n are the strain-hardening strength and strain-hardening exponent. The strain rate term is represented by $1+C\ln\varepsilon^{\cdot *}$, in which the strain rate constant, C, is interrelated with the strain rate. Finally, the softening term is represented by $1-T^{*}{}^{m}$, in that a softening constant, m, is associated with temperature. The $\varepsilon^{\cdot *}$ represents a dimensionless value of the strain rate, in which the strain rate, $\varepsilon^{\cdot }$, is scaled to a reference strain rate, $\varepsilon_{{}^{\circ}}^{\cdot }$, so $\varepsilon^{\cdot *}=\varepsilon^{\cdot }/\varepsilon_{{}^{\circ}}^{\cdot }$. $T^{*}$ is a dimensionless temperature term introduced by $\left(T-T_{r}\right)/\left(T_{m}-T_{r}\right)$, in which T, $T_{r}$, and $T_{m}$ are the tested, reference, and melting temperatures.

To determine the JC constants, reference values for both the strain rate and temperature must be chosen at the beginning. In both reference values, Equation (1) reduces to

(2) $$\sigma =A+B\varepsilon^{n}$$

Taking the logarithm after performing some rearrangements, Equation (2) can be linearly expressed as

(3) $$\ln\left(\sigma -A\right)=\ln\left(B\right)+n\;\ln\left(\varepsilon \right)$$

The value of constant B is obtained from the intercept, and n is the slope of the equation, which is obtained by plotting $\ln\left(\sigma -A\right)$ vs. $\ln\left(\varepsilon \right)$. At the reference temperature, Equation (1) becomes

(4) $$\sigma =\left(A+B\varepsilon^{n}\right)\left(1+C\ln\varepsilon^{\cdot *}\right)$$

After performing some rearrangements, Equation (4) can be linearly expressed as

(5) $$\frac{\sigma }{A+B\varepsilon^{n}}=1+C\ln\varepsilon^{\cdot *}$$

Constant C is the slope of the equation, in that it is obtained by plotting $\sigma /\left(A+B\varepsilon^{n}\right)$ vs. $\ln\left(\varepsilon^{\cdot *}\right)$. To accurately compute the strain rate sensitivity of coefficient C in the JC model, it is recommended that the ratio between every two following strain rates is not less than 0.1 [160], since the exponent, C, has to be calculated from the curves of the logarithmic graph of the true stress vs. true plastic strain for different strain rates.

By taking the logarithm after performing some rearrangements, at reference strain rate, Equation (1) can be written as

(6) $$\ln\left[1-\frac{\sigma }{A+B\varepsilon^{n}}\right]=m\;T^{*}$$

Constant m is the slope of the equation, which is obtained by plotting $\ln\left[1-\sigma /\left(A+B\varepsilon^{n}\right)\right]$ vs. $\;T^{*}$.

**Figure 8:**
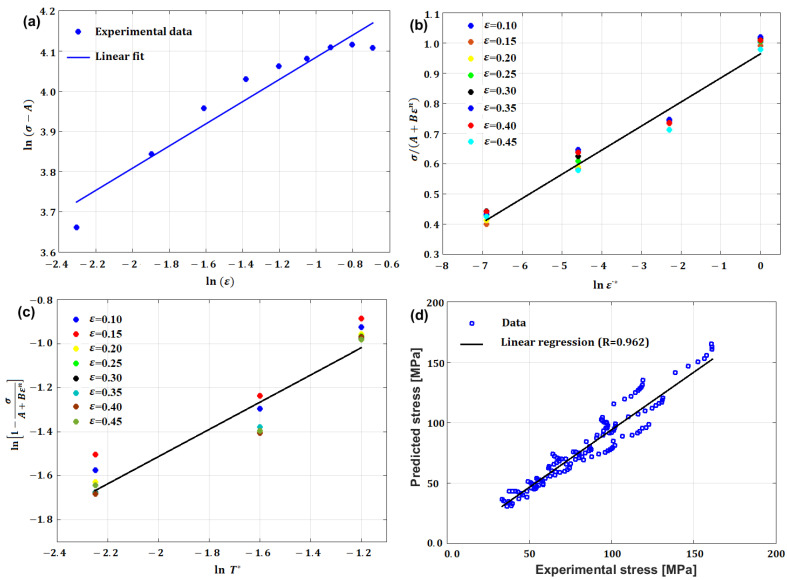
Determination of JC model constants for T24 steel alloy: (**a**) B and n; (**b**) C; (**c**) m; (**d**) correlation between experimental stresses and predicted stresses using JC model.

Li et al. [80] determined the JC constants for T24 steel alloy at elevated temperatures and different strain rates. Figure 8 shows the determination of JC constants for the T24 steel alloy, in which A is the yield stress, which has been found to have a value of 100 MPa, and lnB=4.3567, which provides a B of 78 MPa, while n has the value of the slope, which is found to be 0.2742 (cf. Figure 8a). Constant C is determined as 0.08 (cf. Figure 8b), while constant m has a value of 0.5847 (cf. Figure 8c). The ability of the JC model to accurately predict the flow behavior of T24 steel alloy is assessed using the correlation coefficient (R), which provides a value of 0.962 (cf. Figure 8d).

**Figure 9:**
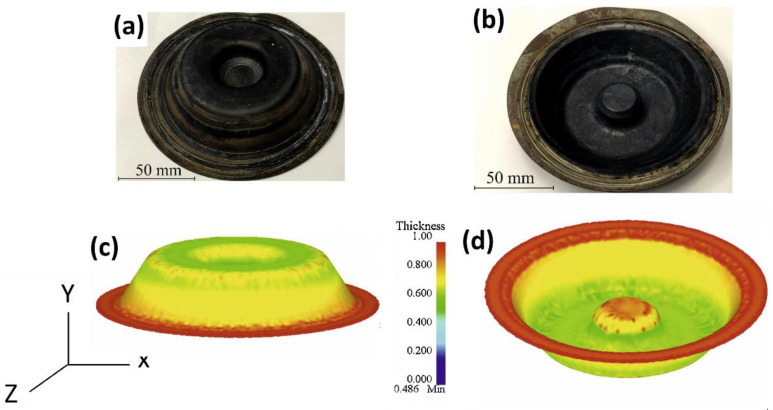
Comparison between experimental superplastic-forming (**a**,**b**) and results of FES (**c**,**d**) for Ti-6%Al-4%V titanium alloy [161].

One of the most powerful advantages of the JC model is that it is implemented in finite element software packages, in which it can be used to mimic real hot working processes and applications in severe conditions using simulation analysis. Mosleh et al. [161] implemented the JC model and two other models with finite element simulations (FES) to optimize the superplastic-forming of Ti-6%Al-4%V titanium alloy. Using FES, the optimized hot working parameters, strain rate, and temperate were determined as 0.002 s^−1^ and 875 °C. The FES was also helpful in checking the behavior of the superplastic-forming of the tested alloy (cf. Figure 9). Many other simulation analyses have been achieved based on the JC model [162,163,164,165,166,167,168].

### 3.2. Modified Johnson–Cook-Based Models

Regardless of the simplicity of the JC model, which contains only five constants, the three parts that constitute the model are independently connected. Many criticisms have been reported for the JC model, since strain hardening, strain rate, and softening are interrelated. In reality, especially with the more complex non-linear behavior of flow stress, hot working parameters, i.e., strain, strain rate, and temperature, are connected [73,76,84,85,90,96,97,169]. Therefore, many modified JC-based models have been introduced in order to precisely predict flow behavior. In the following subsections, $\sigma ,\varepsilon ,\varepsilon^{\cdot },\varepsilon_{0}^{\cdot },\varepsilon^{\cdot *},T^{*},T$, $T_{r}$, and $T_{m}$ are defined as they were used and explained in the original JC model (see Section 3.1).

#### 3.2.1. Meyers JC-Based Modification

Meyers et al. [67] modified the original JC model by replacing the softening term with an exponential term that depends on the temperature rise. The modified model that was presented by Meyers et al. [67] can be expressed as

(7) $$\sigma =\left(A+B\varepsilon^{n}\right)\left(1+C\ln\varepsilon^{\cdot *}\right)e^{-\lambda \left(T-T_{r}\right)}$$

where A, B, n, C,  and λ are material constants. Constants A, B, n, and C are determined in the same way that was explained for the original JC model (see Section 3.1). Taking the logarithm after performing some rearrangements, at different values of the strain rate, Equation (7) can be written as

(8) $$\ln\left[\frac{\sigma }{\left(A+B\varepsilon^{n}\right)\left(1+C\ln\varepsilon^{\cdot *}\right)}\right]=-\lambda \left(T-T_{r}\right)$$

By plotting $\ln\sigma /\left[\left(A+B\varepsilon^{n}\right)\left(1+C\ln\varepsilon^{\cdot *}\right)\right]$ vs. $T-T_{r}$, different values of constant λ can be obtained as the slope of the equation. The average of these values can be taken to determine the value of constant λ.

#### 3.2.2. Rule JC-Based Modification

Rule and Jones [68] presented a modification for the original JC model to capture the sudden rise in strength at very high strain rates (over 10^4^ s^−1^). This modified JC model was applied to OFHC copper, 7075-T6 aluminum, wrought iron, and high-strength steel. The modified JC model that was introduced by Rule and Jones [68] can be expressed as

(9) $$\sigma =\left(C_{1}+C_{2}\varepsilon^{N}\right)\left(1+C_{3}\ln\varepsilon^{\cdot *}+C_{4}\left(\frac{1}{C_{5}-\ln\varepsilon^{\cdot *}}-\frac{1}{C_{5}}\right)\right)\left(1-T^{*}{}^{M}\right)$$

where $C_{1},C_{2},N,C_{3},C_{4},C_{5},\;$ and M are material constants. Rule and Jones [68] modified the well-known finite element code known as elastic–plastic impact computation (EPIC) and determined the whole material’s constants (N,M and $C_{1}-C_{5}$) at once.

**Figure 10:**
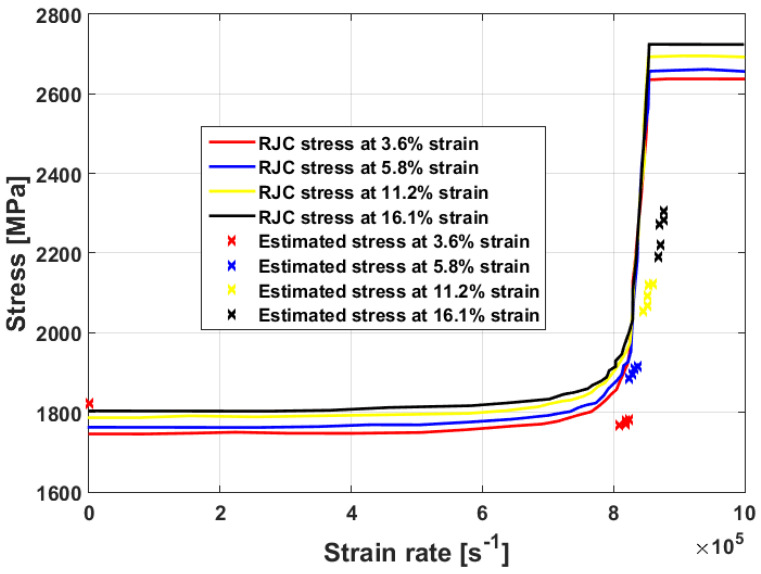
Predicted stresses obtained using the modified JC (RJC) model that was presented by Rule and Jones [68] compared to yield strength in the quasi-static experiment and anticipated from a one-dimensional Taylor specimen model at very high strain rates.

The predicted stresses obtained using the modified JC model that was presented by Rule and Jones [68] are compared to yield strength at different strain rate values, obtained using a quasi-static experiment, and yield strength anticipated from a one-dimensional Taylor specimen model at very high strain rates for the tested high-strength steel (cf. Figure 10). As can be seen, a good agreement is obtained.

#### 3.2.3. Kang JC-Based Modification

Kang et al. [69] modified the strain rate term in the original JC model to predict the dynamic behavior of a vehicle body at very high strain rates. The modified JC model that was presented by Kang et al. [69] can be expressed as

(10) $$\sigma =\left(A+B\varepsilon^{n}\right)\left(1+C_{1}\ln\varepsilon^{\cdot *}+C_{2}{\left(\ln\varepsilon^{\cdot *}\right)}^{2}\right)\left(1-T^{*}{}^{m}\right)$$

where $A,B,n,C_{1},C_{2},\;$ and m are material constants. Constants A,B,n, and m are determined in the same way that was explained for the original JC model (see Section 3.1). At the reference temperature, after performing some rearrangements, Equation (10) turns into

(11) $$\frac{\sigma }{\left(A+B\varepsilon^{n}\right)}=1+C_{1}\ln\varepsilon^{\cdot *}+C_{2}{\left(\ln\varepsilon^{\cdot *}\right)}^{2}$$

**Figure 11:**
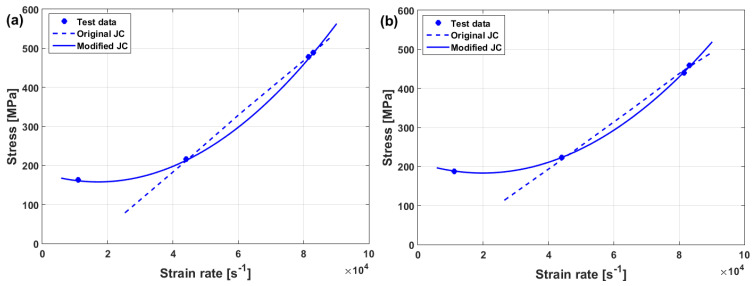
Initial yield stress vs. strain rate using both the original JC model and the modified JC model that was presented by Kang et al. [69] for (**a**) SPCC and (**b**) SPRC.

Figure 11 shows the initial yield stress vs. the strain rate for both the original JC and the modified JC that was presented by Kang et al. [69] for steel plate cold-rolled with grade C (SPCC) (cf. Figure 11a) and steel plate rolled with steel grade C (SPRC) (cf. Figure 11b). The figure shows that quadratic fitting can be used with the modified JC model. Similar findings can be obtained using the modified JC model introduced by Kang et al. [69] to investigate the dynamic behavior of autobody sheet metal [70]. Consequently, by plotting $\sigma /\left(A+B\varepsilon^{n}\right)$ vs. $\ln\varepsilon^{\cdot *}$, constants $C_{1}$ and $C_{2}$ can be determined with quadratic fitting.

#### 3.2.4. Couque JC-Based Modification

Couque et al. [71] presented another modification for the strain rate term in the original JC model, in which a power constant is correlated with the strain rate. The modified JC model that was presented by Couque et al. [71] can be written as

(12) $$\sigma =\left(A+B\varepsilon^{n}\right)\left(1+C_{1}\ln\frac{\varepsilon^{\cdot }}{\varepsilon^{\cdot }{}_{0}}+E{\left(\frac{\varepsilon^{\cdot }}{\varepsilon^{\cdot }{}_{1}}\right)}^{k}\right)\left(1-T^{*}{}^{m}\right)$$

where $\;A,B,n,C_{1},k$, and m are material constants. The strain rate, $\varepsilon^{\cdot }{}_{1}$, defines a transition between thermally activated and viscous regimes and is stated to have 0.001 s^−1^ [71]. Constants A,B,n, and m are determined in the same way that was explained for the original JC model (see Section 3.1). At the reference temperature, after performing some rearrangements, Equation (12) can be written as

(13) $$\frac{\sigma }{\left(A+B\varepsilon^{n}\right)}=1+C_{1}\ln\frac{\varepsilon^{\cdot }}{\varepsilon^{\cdot }{}_{0}}+E{\left(\frac{\varepsilon^{\cdot }}{\varepsilon^{\cdot }{}_{1}}\right)}^{k}$$

**Figure 12:**
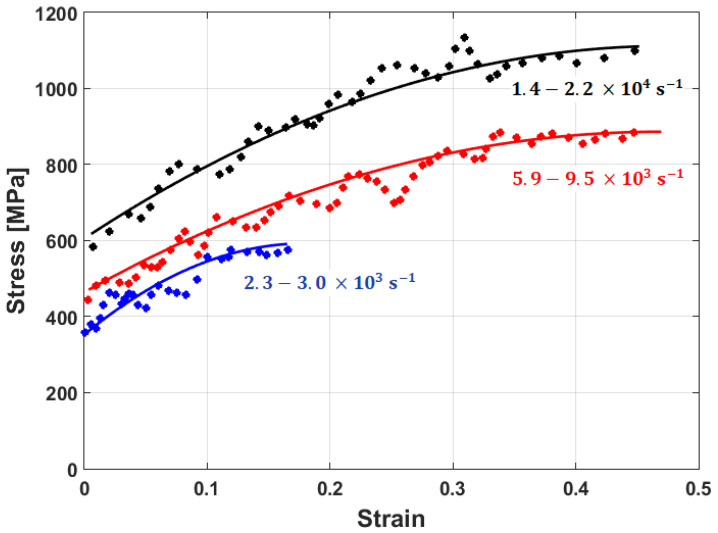
Experimental stresses (markers) compared to predicted stresses (solid lines) using the modified JC model that was presented by Couque et al. [71] for nickel.

Constants $C_{1},E$, and k can be determined using algorithms that are based on non-linear least square methods. Good fitting between experimental stresses and predicted stresses are obtained using the modified JC method that was presented by Couque et al. [71] for the prediction of flow stress in nickel at different strain rates, and the home temperature can be obtained (cf. Figure 12).

A similar modification for the strain rate term is presented by Johnson et al. [72], in which the strain rate term is introduced by

$$1+C_{1}\ln\frac{\varepsilon^{\cdot }}{\varepsilon^{\cdot }{}_{0}}+C_{2}{\left(\ln\frac{\varepsilon^{\cdot }}{\varepsilon^{\cdot }{}_{0}}\right)}^{C_{3}}$$

where constants $C_{1},C_{2}$, and $C_{3}$ can be determined by using the exponential fitting of $\sigma /\left(A+B\varepsilon^{n}\right)$ vs. $\ln\varepsilon^{\cdot *}$.

#### 3.2.5. Lin (1) JC-Based Modification

Lin et al. [73] introduced one of the famous modifications of the original JC model to study the flow behavior of typical high-strength alloy steel. The coupling effect between the hot working parameters, strain, strain rate, and temperature is taken into consideration in the proposed modification. The modified JC model that was introduced by Lin et al. [73] can be expressed as

(14) $$\sigma =\left(A+B_{1}\varepsilon +B_{2}\varepsilon^{2}\right)\left(1+C_{1}\ln\varepsilon^{\cdot *}\right)\exp\left[\left(\lambda_{1}+\lambda_{2}\ln\varepsilon^{\cdot *}\right)\left(T-T_{r}\right)\right]$$

where $A,\;B_{1},\;B_{2},\;C_{1},\;\lambda_{1},\;$ and $\lambda_{2}$ are material constants. As can be seen from Equation (14), the first term in the JC model is replaced with a quadratic function of strain, with constants $A,\;B_{1}$, and $B_{2}$. In this modification, the strain rate term in the original JC is employed as it is, with constant $C_{1}$. The third term in the original JC model is modified considering the coupling effect between the strain rate and temperature considering two constants, $\lambda_{1}\;$ and $\lambda_{2}$.

**Figure 13:**
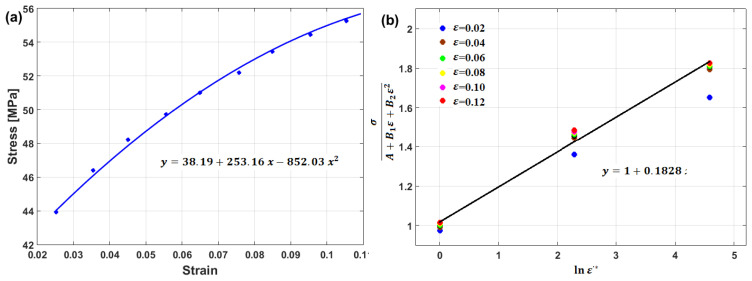
Determination of the modified JC model constants: (**a**) $A,\;B_{1}$, and $B_{2}$ and (**b**) $C_{1}$.

At the reference strain rate and reference temperature, Equation (14) reduces to

(15) $$\sigma =A+B_{1}\varepsilon +B_{2}\varepsilon^{2}$$

where constants $A,\;B_{1}$, and $B_{2}$ are determined by fitting the experimental data of σ vs. ε at the reference values with the quadratic function in the strain (cf. Figure 13a). At the reference temperature, Equation (14) lowers to

(16) $$\sigma =\left(A+B_{1}\varepsilon +B_{2}\varepsilon^{2}\right)\left(1+C_{1}\ln\varepsilon^{\cdot *}\right)$$

After performing some rearrangements, Equation (16) can be written as

(17) $$\frac{\sigma }{A+B_{1}\varepsilon +B_{2}\varepsilon^{2}}=1+C_{1}\ln\varepsilon^{\cdot *}$$

Strain rate constant $C_{1}$ is the slope of Equation (17) and is obtained by plotting $\sigma /\left(A+B_{1}\varepsilon +B_{2}\varepsilon^{2}\right)$ vs. $\ln\varepsilon^{\cdot *}$ (cf. Figure 13b).

To obtain the value of the two constants, $\lambda_{1}$ and $\lambda_{2}$, a new parameter, λ, that is equal to $\lambda_{1}+\lambda_{2}\ln\varepsilon^{\cdot *}$ is introduced, after performing some rearrangements, Equation (14) can be expressed as

(18) $$\frac{\sigma }{\left(A+B_{1}\varepsilon +B_{2}\varepsilon^{2}\right)\left(1+C_{1}\ln\varepsilon^{\cdot *}\right)}=e^{\lambda \left(T-T_{r}\right)}$$

By taking the logarithm of both sides, Equation (18) can be written as

(19) $$\ln\left[\frac{\sigma }{\left(A+B_{1}\varepsilon +B_{2}\varepsilon^{2}\right)\left(1+C_{1}\ln\varepsilon^{\cdot *}\right)}\right]=\lambda \left(T-T_{r}\right)$$

**Figure 14:**
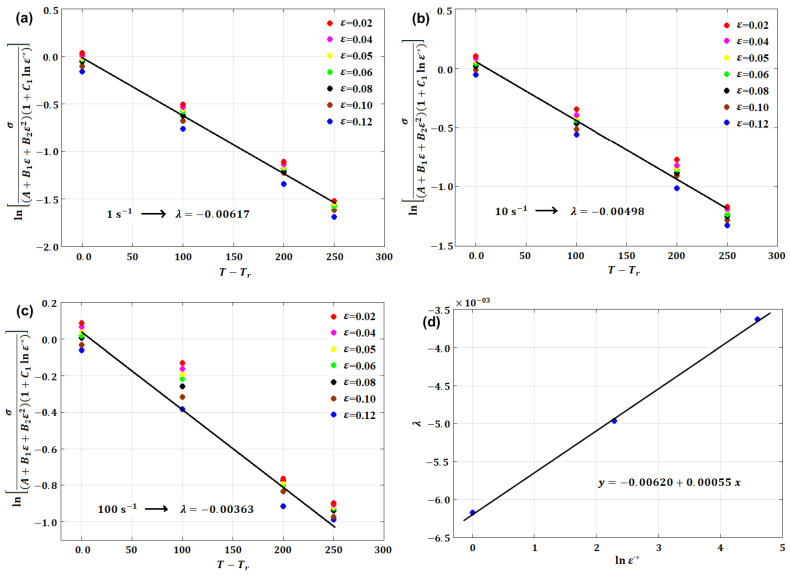
Determination of the modified JC constants $\lambda ,\lambda_{1}$, and $\lambda_{2}$: (**a**) λ at 1 s^−1^; (**b**) λ at 10 s^−1^; (**c**) λ at 100 s^−1^; (**d**) $\lambda_{1}$ and $\lambda_{2}$.

By plotting $\ln\left[\sigma /\left(\left(A+B_{1}\varepsilon +B_{2}\varepsilon^{2}\right)\left(1+C_{1}\ln\varepsilon^{\cdot *}\right)\right)\right]$ vs. $\left(T-T_{r}\right)$ at different strain rate and temperature values, different values for λ (slope of the equation; cf. Figure 14a–c) are obtained, which can be plotted vs. $\ln\varepsilon^{\cdot *}$, in which the value of $\lambda_{1}$ is the intercept and the value of $\lambda_{2}$ is the slope (Figure 14d).

**Figure 15:**
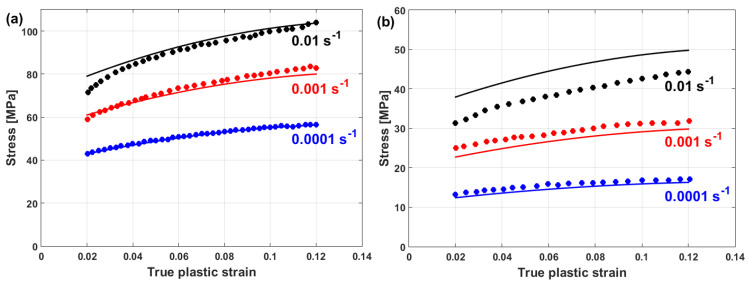
Experimental stresses (markers) compared to predicted stresses (solid lines) obtained by the modified JC model that was presented by Lin et al. [73] for typical high-strength alloy steel at (**a**) 1123 K and (**b**) 1323 K.

Experimental stresses are compared to predicted stresses that were obtained by the modified JC model that was introduced by Lin et al. [73] for typical high-strength alloy steel at different strain rates and different temperatures, as shown in Figure 15. The figure shows that the predicted stresses agree very well with the experimental stresses, with a maximum relative error (RE) of 5.15%. The modified JC model that was introduced by Lin et al. [73] has been used with many different alloys [170,171,172,173,174], with accurate predictions and inaccurate predictions for other alloys [61,96,175,176,177].

#### 3.2.6. Hou Q. Y. JC-Based Modification

Hou Q. Y. et al. [74] modified the softening term in the original JC model, considering that the tested temperature might be higher or lower than the reference temperature. The modified JC model that was presented by Hou Q. Y. et al. [74] can be expressed as

(20) $$\sigma =\left(A+B\varepsilon^{n}\right)\left(1+C\ln\varepsilon^{\cdot *}\right)\left(1-\lambda \frac{e^{T/T_{m}}-e^{T_{r}/T_{m}}}{e-e^{T_{r}/T_{m}}}\right)$$

where constants A, B, n, and C are determined as was explained for the original JC model (see Section 3.1). At different strain rate values, after performing some rearrangements, Equation (20) can be written as

(21) $$1-\frac{\sigma }{\left(A+B\varepsilon^{n}\right)}=\lambda \frac{e^{T/T_{m}}-e^{T_{r}/T_{m}}}{e-e^{T_{r}/T_{m}}}$$

By plotting $1-\sigma /\left(A+B\varepsilon^{n}\right)$ vs. $\left(e^{T/T_{m}}-e^{T_{r}/T_{m}}\right)/\left(e-e^{T_{r}/T_{m}}\right)$, at different values of strain, different values of constant λ (slope of the equation) can be obtained, in which the average can be implemented.

**Figure 16:**
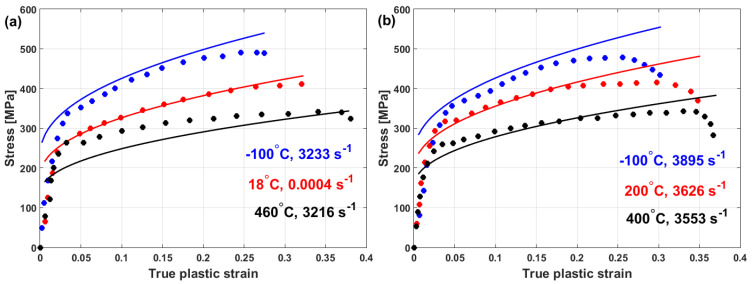
Experimental stresses (markers) compared to predicted stresses (solid lines) obtained by the modified JC that was presented by Hou Q. Y. et al. [74] for Mg–10Gd–2Y–0.5Zr alloy with the following dimensions: (**a**) Փ 10 mm × 5 mm and (**b**) Փ 10 mm × 4 mm.

A good agreement between experimental stresses and predicted stresses obtained by the modified JC that was presented by Hou Q. Y. et al. [74] for Mg–10Gd–2Y–0.5Zr alloy is achieved (cf. Figure 16).

**Figure 17:**
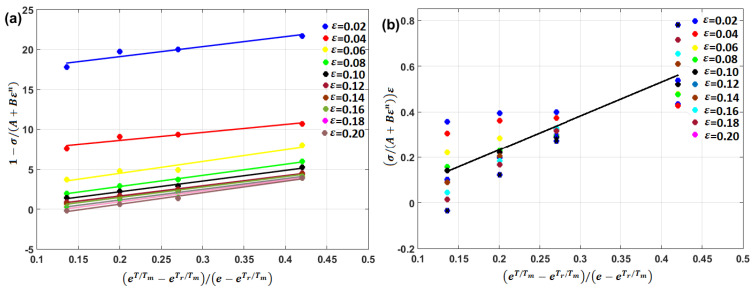
Determination of (**a**) λ and (**b**) $\lambda^{\prime }$ using regression analysis.

Perez et al. [178] used the modified JC that was introduced by Hou Q. Y. et al. [74] for the prediction of the flow behavior of Ti6Al4V alloy at high temperatures. The modified model succeeded in predicting the flow behavior of the tested titanium alloy with an R of 0.9765 compared with other models. In fact, considering Equation (21), λ varies with strain, and, hence, Guoliang et al. [179] modified Equation (21) by replacing λ with λ′ε, in that $\lambda^{\prime }$ has a constant value. Figure 17 shows the regression of both λ and $\lambda^{\prime }$ by plotting $\left(e^{T/T_{m}}-e^{T_{r}/T_{m}}\right)/\left(e-e^{T_{r}/T_{m}}\right)$ vs. $1-\sigma /\left(A+B\varepsilon^{n}\right)$ and $\left(e^{T/T_{m}}-e^{T_{r}/T_{m}}\right)/\left(e-e^{T_{r}/T_{m}}\right)$ vs. $\left(\sigma /\left(A+B\varepsilon^{n}\right)\right)\varepsilon$, respectively, which was performed by Guoliang et al. [179].

#### 3.2.7. Shin JC-Based Modification

**Figure 18:**
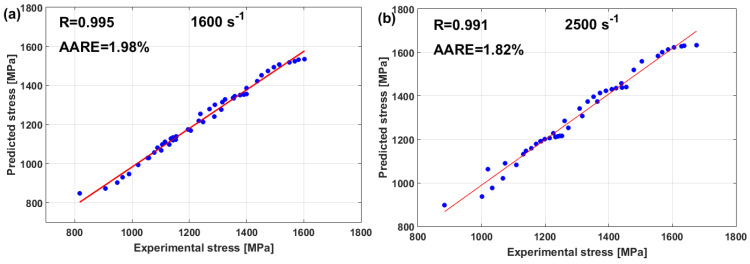
Correlation of experimental stresses and predicted stresses obtained by the modified JC model that was presented by Shin and Kim [75] for tungsten heavy alloy at 298 K, 773 K, and 1173 K for (**a**) 1600 s^−1^ and (**b**) 2500 s^−1^.

Shin and Kim [75] modified the original JC model for the accurate prediction of materials at wide regimes of temperature and strain rate. The well-known Voce hardening [180] is implemented in the modified JC model since it is describing the saturation of strain hardening. Thus, the strain rate term is also modified, so the high increase in the stress due to large strain rates can be taken into consideration. Furthermore, the temperature softening constant m is applied to the whole bracket instead of $T^{*}$. The modified JC model that was presented by Shin and Kim [75] can be expressed as

(22) $$\sigma =\left[A+B\left\{1-\exp\left(-C\varepsilon \right)\right\}\right]\left[D\;\ln\left(\varepsilon^{\cdot *}\right)+\exp\left(E\;\varepsilon^{\cdot *}\right)\right]{\left[1-T^{*}\right]}^{m}$$

where constants A and B represent yield and saturated stress, constant C represents the strain-hardening exponent, and constants D and E correlate to the strain rate, while constant m correlates to softening. At the reference strain rate and reference temperature, the Voce hardening is the only left term in Equation (22), as $\sigma =\left[A+B\left\{1-\exp\left(-C\varepsilon \right)\right\}\right]$, in which A can be measured as the yield stress, and B and C can be computed using a regression analysis that is based on the non-linear least square method. Similarly, constants D, E, and m can be determined. Figure 18 shows that the predicted stresses of the modified JC model that was introduced by Shin and Kim [75] have a very good agreement with the experimental stresses for tungsten heavy alloy at high temperatures and different strain rates, with R = 0.995 and AARE = 1.98% (cf. Figure 18a) and with R = 0.991 and AARE = 1.82% (cf. Figure 18b).

**Figure 19:**
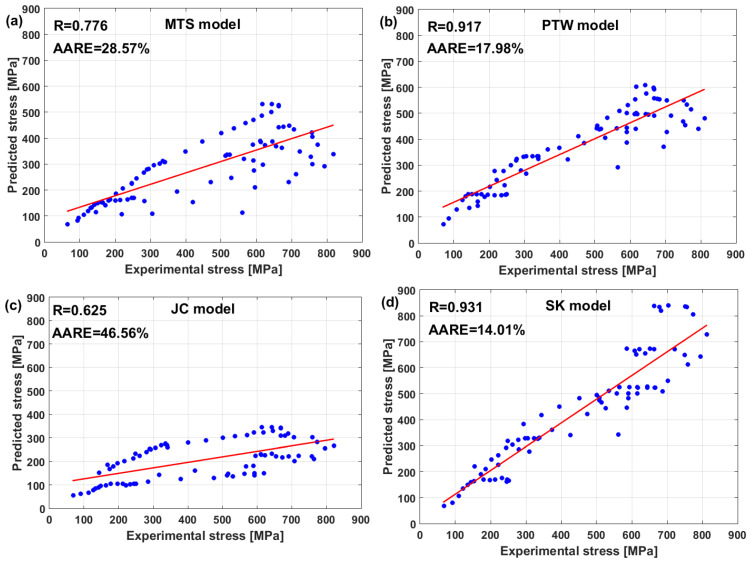
Correlation of experimental stresses and predicted stresses for copper at temperatures of 571–1096 K and strain rates of 4000–693,000 s^−1^ obtained by (**a**) the MTS model (**b**), PTW model (**c**), JC model, and (**d**) SK model.

In another published article, Shin and Kim [181] studied the capability of the Shin and Kim [75] modified JC model for the prediction of the flow behavior of copper at a wide range of strain rates and temperatures. Compared with the original JC model, the mechanical threshold (MTS) model, and the Preston–Tonks–Wallace (PTW) model, the Shin and Kim (SK) model [75] provided the best predictions for the flow behavior of copper, with R = 0.931 and AARE = 14.01%, as shown in Figure 19.

#### 3.2.8. Maheshwari JC-Based Modification

Maheshwari et al. [76] modified the original JC material flow model during hot deformation. The modified JC model can be expressed as

(23) $$\sigma =\left(P+Q\varepsilon^{n}\right){\left(\frac{\varepsilon^{\cdot }}{\varepsilon_{{}^{\circ}}^{\cdot }}\right)}^{r}\left[1+\left\{\frac{\sigma_{m}}{\sigma_{y}}-1\right\}\exp\left\{-\alpha {\left(\frac{T_{m}-T}{T-T_{r}}\right)}^{\beta }\right\}\right]$$

where $\sigma_{y}$ is reference stress, and $\sigma_{m}$ is the true stress upon melting, which has a reference value of zero. Constants P, Q, n, r, α, and β are six material constants that constitute the modified model. At the reference strain rate and reference temperature, Equation (23) lowers to $\sigma =P+Q\varepsilon^{n},\;$ with P equal to yield stress. Constants Q  and n can be determined by fitting the obtained linear logarithmic equation, as explained in the original JC model (see Section 3.1). Taking logarithms for both sides of Equation (23), after performing some rearrangements, and at a reference temperature, the equation can be expressed as

(24) $$\ln\left[\frac{\sigma }{P+Q\varepsilon^{n}}\right]=r\ln\left(\frac{\varepsilon^{\cdot }}{\varepsilon_{{}^{\circ}}^{\cdot }}\right)$$

By plotting $\ln\left[\sigma /\left(P+Q\varepsilon^{n}\right)\right]$ vs. $\ln\left(\varepsilon^{\cdot }/\varepsilon_{{}^{\circ}}^{\cdot }\right)$, constant r has the value of the slope. Finally, at different strain rate and temperature values, after performing some rearrangement and taking logarithms for both sides, Equation (23) can be expressed as

(25) $$\ln\left[\frac{\sigma }{\left(P+Q\varepsilon^{n}\right){\left(\varepsilon^{\cdot }/\varepsilon_{{}^{\circ}}^{\cdot }\right)}^{r}}\right]=-\alpha {\left(\frac{T_{m}-T}{T-T_{r}}\right)}^{\beta }$$

By plotting $\ln\left[\sigma /\left(\left(P+Q\varepsilon^{n}\right){\left(\varepsilon^{\cdot }/\varepsilon_{{}^{\circ}}^{\cdot }\right)}^{r}\right)\right]$ vs. $\left(T_{m}-T\right)/\left(T-T_{r}\right)$, the two constants α and β can be computed by fitting the data with an exponential function.

**Figure 20:**
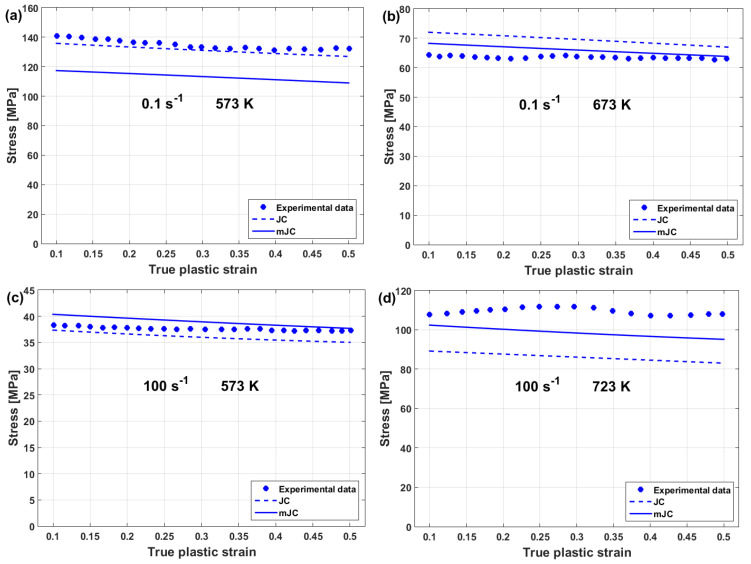
Comparison of experimental stresses and predicted stresses obtained by JC and the modified JC that was presented by Maheshwari et al. [76] for Al-2024 alloy at (**a**) 0.1 s^−1^ and 573 K, (**b**) 0.1 s^−1^ and 673 K, (**c**) 100 s^−1^ and 573 K, and (**d**) 100 s^−1^ and 723 K.

Compared with the original JC model, the modified JC that was introduced by Maheshwari et al. [76] gave accurate predictions for some of the strain rate and temperature combinations, while it gave inaccurate predictions with other combinations. A comparison between experimental stresses and predicted stresses obtained by the original JC and by the modified JC that was presented by Maheshwari et al. [76] for Al-2024 alloy under hot deformation is shown in Figure 20. As can be seen, the modified model cannot guarantee precise predictions of stresses.

The predictability of the flow behavior using the modified JC that is presented by Maheshwari et al. [76], along with other models, was investigated for 2024Al alloy under hot deformations by Trimble and O’Donnell [182]. The results showed that the model provides good predictions of the flow behavior of the tested alloy, with an R of 0.992 and an average absolute relative error (AARE) of 5.9491%. However, it failed to accurately predict the flow behavior in some combinations between the strain rate and temperature. The same findings were obtained by Maheshwari [183].

#### 3.2.9. Wang (1) JC-Based Modification

Wang et al. [77] modified the JC model to predict the flow behavior of 30Cr2Ni4MoV rotor steel alloy through a large range of strain rates and temperatures. The coupling effect between the strain, strain rate, and temperature is taken into account in this modification. In this modification, the Voce hardening [180] equation is used to describe the strain-hardening term. The modified JC model that was presented by Wang et al. [77] can be written as

(26) $$\sigma =\left[A-B_{0}\exp\left(-B_{1}\varepsilon \right)\right]\left[1+\left(C_{1}+C_{2}\varepsilon \right)\;\ln\left(\varepsilon^{\cdot *}\right)\right]\exp\left[\left(\lambda_{1}+\lambda_{2}\ln\varepsilon^{\cdot *}\right)\left(T-T_{L}\right)\right]$$

where $T_{L}$ is defined as the lowest temperature in the tested range of temperatures, and $A,\;B_{0},\;B_{1},\;C_{1},\;C_{2},\;\lambda_{1},\;$ and $\lambda_{2}$ are the material constants. At the reference strain rate and lowest temperature, Equation (26) lowers to $\sigma =\left[A-B_{0}\exp\left(-B_{1}\varepsilon \right)\right]$. Taking into account that A represents the yield stress, constants $B_{0}$ and $B_{1}$ can be determined using regression analysis by plotting σ vs. ε. At the lowest temperature, after performing some rearrangements with $C=C_{1}+C_{2}\varepsilon$, Equation (26) can be expressed as

(27) $$\frac{\sigma }{\left[A-B_{0}\exp\left(-B_{1}\varepsilon \right)\right]}=\left[1+C\;\ln\left(\varepsilon^{\cdot *}\right)\right]$$

**Figure 21:**
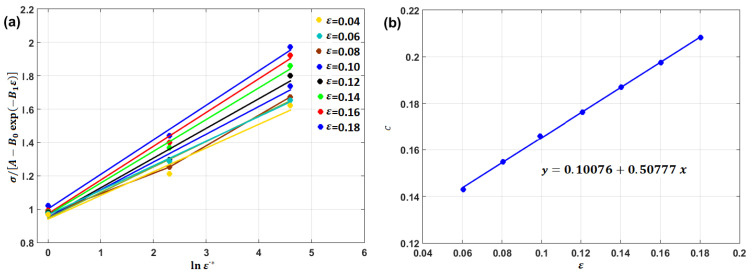
Determination of $C_{1}$ and $C_{2}$: (**a**) $\sigma /\left[A-B_{0}exp\left(-B_{1}\varepsilon \right)\right]$ vs. $ln\;\left(\varepsilon^{\cdot *}\right)$ to obtain C values and (**b**) C vs. ε to obtain $C_{1}$ and $C_{2}$.

By plotting $\sigma /\left[A-B_{0}\exp\left(-B_{1}\varepsilon \right)\right]$ vs. $\ln\left(\varepsilon^{\cdot *}\right)$ at different strain and strain rate values, different values of C can be obtained (cf. Figure 21a). Hence, C can be linearly plotted vs. ε, in which the value of $C_{1}$ is the intercept and the value of $C_{2}$ is the slope (cf. Figure 21b).

At different strain rate values, after performing some rearrangements, Equation (26) can be written as

(28) $$\frac{\sigma }{\left[A-B_{0}\exp\left(-B_{1}\varepsilon \right)\right]\left[1+C\;\ln\left(\varepsilon^{\cdot *}\right)\right]}=\exp\left[\left(\lambda_{1}+\lambda_{2}\ln\varepsilon^{\cdot *}\right)\left(T-T_{L}\right)\right]$$

Taking the logarithm of both sides and introducing a new parameter, λ = $\lambda_{1}+\lambda_{2}\ln\varepsilon^{\cdot *}$, the equation turned out to be linear with a slope of λ. The two constants, $\lambda_{1}$ and $\lambda_{2}$, can be determined as explained in Section 3.2.5.

**Figure 22:**
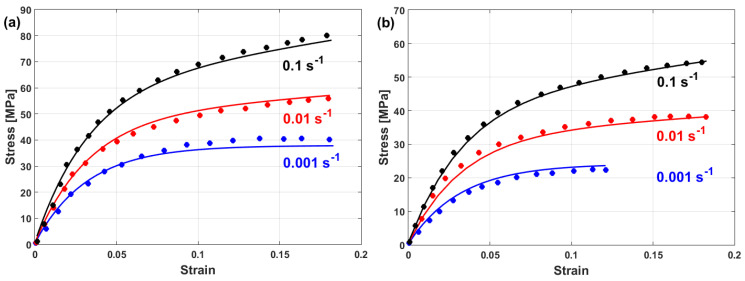
Experimental stresses (markers) compared to predicted stresses (solid lines) using the modified JC that was presented by Wang et al. [77] for 30Cr2Ni4MoV rotor steel at (**a**) 1323 K and (**b**) 1423 K.

A comparison between experimental stresses and predicted stresses obtained using the modified JC model that was presented by Wang et al. [77] for 30Cr2Ni4MoV rotor steel alloy at elevated temperatures and different strain rates is shown in Figure 22. As can be seen, the predicted stresses obtained by the modified JC model have very good agreements with the experimental stresses. This might be due to the use of Voce hardening, as well as taking the coupling effect between the strain rate and temperature into consideration.

#### 3.2.10. Lin (2) JC-Based Modification

Lin et al. [78] modified the original JC model to predict the flow behavior of Al-Zn-Mg-Cu alloy under hot deformation. The modification considers the coupling effect of the strain, strain rate, and temperature. The modified JC model that was presented by Lin et al. [78] can be written as

(29) $$\sigma =\left(\sigma_{0}+B\left(\varepsilon^{\cdot }\right)\varepsilon^{n\left(\varepsilon^{\cdot }\right)}\right)\left[1-T^{*p\left(\varepsilon^{\cdot }\right)}\right]$$

In this modification, and in order to overcome the difficulty of finding yield stress, the yield stress is replaced by beak stress and defined using an Arrhenius-type equation as per [78]:

(30) $$\sigma_{0}=\frac{1}{\alpha }\ln\left\{{\left(\frac{Z}{A}\right)}^{1/m}+{\left[{\left(\frac{Z}{A}\right)}^{2/m}+1\right]}^{1/2}\right\}$$

where $Z=\varepsilon^{\cdot }\exp\left(Q/RT\right)$; Q is the activation energy; and R is the universal gas constant, which has a value of 8.31 Jmol^−1^·K^−1^. Q, α, A, and m are constants to be determined; see [78] for more information. By taking the logarithm after performing some rearrangements, at the reference temperature, Equation (29) can be expressed as

(31) $$\ln\left[\sigma -\sigma_{0}\right]=\ln B\left(\varepsilon^{\cdot }\right)+n\left(\varepsilon^{\cdot }\right)\ln\varepsilon$$

By plotting $\ln\left[\sigma -\sigma_{0}\right]$ vs. lnε, the parameters of $B\left(\varepsilon^{\cdot }\right)$ can be obtained from the intercept, and the parameters of $n\left(\varepsilon^{\cdot }\right)$ can be obtained from the slope; then, using polynomial fitting for $B\left(\varepsilon^{\cdot }\right)$ with $\varepsilon^{\cdot }$ and for $n\left(\varepsilon^{\cdot }\right)$ with $\varepsilon^{\cdot }$ the constants that constitute their equations can be obtained. By taking the logarithm after performing some rearrangements, at different strain rates, Equation (29) can be written as

(32) $$\ln\left[1-\frac{\sigma }{\sigma_{0}+B\left(\varepsilon^{\cdot }\right)\varepsilon^{n\left(\varepsilon^{\cdot }\right)}}\right]=p\left(\varepsilon^{\cdot }\right)\ln T^{*}$$

At different values for both the temperature and strain rate, different values of $p\left(\varepsilon^{\cdot }\right)$ can be obtained; then, the polynomial fitting of $p\left(\varepsilon^{\cdot }\right)$ with $\varepsilon^{\cdot }$ can be used to determine the parameters of $p\left(\varepsilon^{\cdot }\right)$ (see Section 3.2.5).

Lin et al. [78] fitted $B\left(\varepsilon^{\cdot }\right),\;n\left(\varepsilon^{\cdot }\right)$, and $p\left(\varepsilon^{\cdot }\right)$ vs. $\varepsilon^{\cdot }$ with linear relationships for Zn-Mg-Cu alloy, which can be expressed as $B\left(\varepsilon^{\cdot }\right)=5.69337+8.77317\ln\varepsilon^{\cdot }$, $n\left(\varepsilon^{\cdot }\right)=1.66485+0.10504\ln\varepsilon^{\cdot }$, $p\left(\varepsilon^{\cdot }\right)=0.71291+0.04391\varepsilon^{\cdot }$.

**Figure 23:**
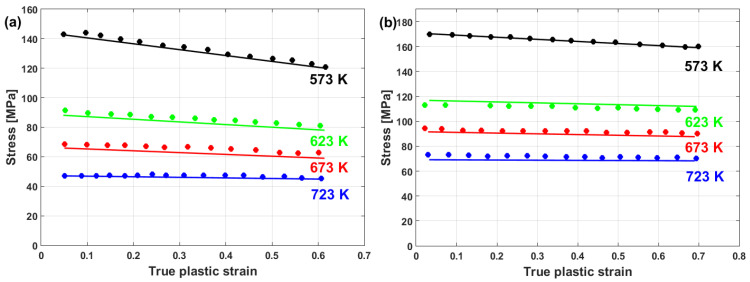
Comparison of experimental stresses (markers) and predicted stresses (solid lines) using the modified JC model that was introduced by Lin et al. [78] for Zn-Mg-Cu alloy at (**a**) 0.005 s^−1^ and (**b**) 0.1 s^−1^.

A comparison between experimental stresses and predicted stresses obtained using the modified JC model that was introduced by Lin et al. [78] for the prediction of Zn-Mg-Cu alloy is shown in Figure 23. As can be seen, very accurate predictions were obtained, which might be due to the coupling effect of strain, strain rate, and temperature that was taken into consideration.

#### 3.2.11. Lin (3) JC-Based Modification

Lin et al. [79] presented another modification of the original JC model to predict the flow behavior of 7075 Al alloy under hot deformation. The modification considers the coupling effect of strain, strain rate, and forming temperature. The modified JC model that was presented by Lin et al. [79] can be written as

(33) $$\sigma =\left(\sigma_{0}+B\left(T\right)\varepsilon^{n\left(T\right)}\right)\left[1+C\left(T\right)\ln\varepsilon^{\cdot *}\right]$$

$\sigma_{0}$ is represented by using an Arrhenius-type equation, as in Section 3.2.10, the same as Equation (30). By following the same procedures in Section 3.2.10, the parameters of $B\left(T\right),\;n\left(T\right)$ and $C\left(T\right)$ can be obtained using fitting analysis.

Lin et al. [79] determined $B\left(T\right),\;n\left(T\right)$, and $C\left(T\right)$ as linear relationships in T for the 7075 Al alloy as $B\left(T\right)=-102.75+1.283\times 10^{-1}T$, $n\left(T\right)=-0.26576+6.2\times 10^{-1}T$, and $C\left(\varepsilon^{\cdot }\right)=-3.44+7.43\times 10^{-1}T$.

**Figure 24:**
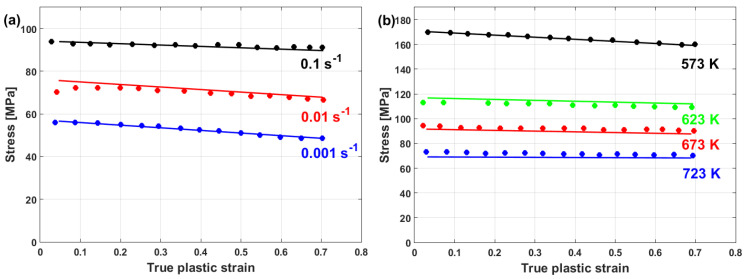
Comparison of experimental stresses (markers) and predicted stresses (solid lines) using the modified JC model that was presented by Lin et al. [79] for 7075 Al alloy at (**a**) 673 K and (**b**) 723 K.

A comparison between the experimental stresses and predicted stresses obtained using the modified JC model that was presented by Lin et al. [79] for the prediction of the flow behavior of 7075 Al alloy is shown in Figure 24. As can be seen, very accurate predictions were obtained, which might be due to the coupling effect of strain, strain rate, and temperature that was taken into consideration.

#### 3.2.12. Li JC-Based Modification

Li et al. [80] introduced a modification of the original JC model for the prediction of the flow behavior of T24 steel at elevated temperatures and different strain rates. In this modification, both strain-hardening and -softening terms are modified, while the strain rate term is kept the same as in the original JC model. At reference values, the experimental stresses vs. strains are fitted to second-order polynomial function, while the softening term takes the coupling effect of strain and temperature, as well as strain rate and temperature into account. The modified JC model that was presented by Li et al. [80] can be expressed as

(34) $$\sigma =\left(A+B_{1}\varepsilon +B_{2}\varepsilon^{2}\right)\left(1+C\ln\varepsilon^{\cdot *}\right)\exp\left[\left(Q\left(\varepsilon \right)+V\left(\varepsilon^{\cdot },\varepsilon \right)\ln\varepsilon^{\cdot *}\right)\left(T-T_{r}\right)\right]$$

where $A,\;B_{1},\;B_{2},\;C,\;Q,\;$ and V are material constants, taking into account that constant  Q  depends on ε, and constant V depends on $\varepsilon^{\cdot }$ and ε. Constants $A,\;B_{1},\;B_{2}$, and C can be determined in the same way that was explained in Section 3.2.5. At the reference strain rate, Equation (34) turns into

(35) $$\sigma =\left(A+B_{1}\varepsilon +B_{2}\varepsilon^{2}\right)\exp\left[\left(Q\left(\varepsilon \right)\right)\left(T-T_{r}\right)\right]$$

Taking logarithms for both sides after performing some rearrangements, Equation (35) can be written as

(36) $$\ln\left[\frac{\sigma }{A+B_{1}\varepsilon +B_{2}\varepsilon^{2}}\right]=\left(Q\left(\varepsilon \right)\right)\left(T-T_{r}\right)$$

The slope of the equation is $Q\left(\varepsilon \right)$; since it depends on the strain, different values for Q can be obtained at different values of strain. Then, a polynomial fit is used to determine Q as a function of ε. Constant $V\left(\varepsilon^{\cdot },\varepsilon \right)$ can be determined by introducing a new parameter, S, which is equal to $Q\left(\varepsilon \right)+V\left(\varepsilon^{\cdot },\varepsilon \right)\ln\varepsilon^{\cdot *}$. After performing some rearrangements, at the remaining values of the strain rates, Equation (34) can be expressed as

(37) $$\frac{\sigma }{\left(A+B_{1}\varepsilon +B_{2}\varepsilon^{2}\right)\left(1+C\ln\varepsilon^{\cdot *}\right)}=\exp\left[S\left(T-T_{r}\right)\right]$$

By taking logarithms for both sides, Equation (37) can be introduced as

(38) $$\ln\left[\frac{\sigma }{\left(A+B_{1}\varepsilon +B_{2}\varepsilon^{2}\right)\left(1+C\ln\varepsilon^{\cdot *}\right)}\right]=S\left(T-T_{r}\right)$$

Different values for the slope, S, are obtained with different values for the temperature, strain, and strain rate; then, different values for $V\left(\varepsilon^{\cdot },\varepsilon \right)$, can be determined as a function of the strain and strain rate by inputting $V\left(\varepsilon^{\cdot },\varepsilon \right)=\left[S-Q\left(\varepsilon \right)\right]/\ln\varepsilon^{\cdot *}$. For T24 steel, Li et al. [80] determined the polynomial function of Q as $-0.00344+0.00177\varepsilon -0.00135\varepsilon^{2}$, while the values of V at different strain and strain rate values are shown in Table 1.

**Figure 25:**
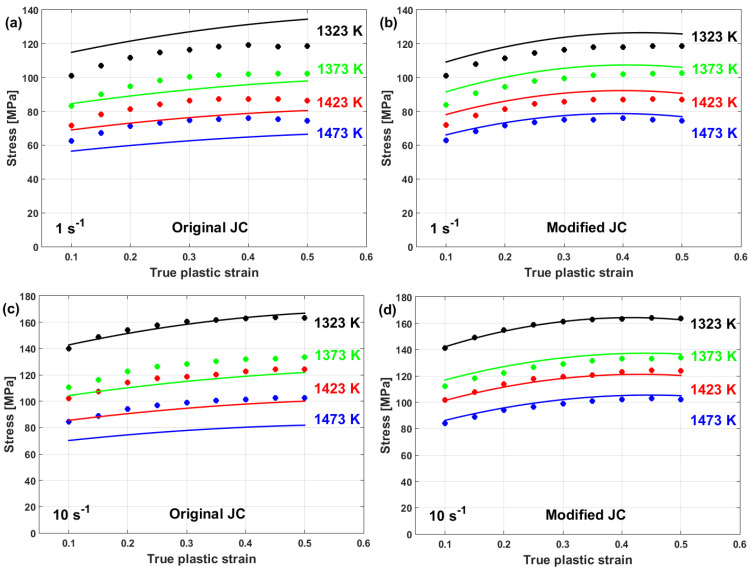
Comparison between experimental stresses (markers) and predicted stresses (solid lines) of T24 steel under hot deformation obtained by (**a**,**c**) the original JC model and (**b**,**d**) the modified JC model that was presented by Li et al. [80].

Good predictability for the prediction of the flow stress of T24 steel using the modified JC model that was presented by Li et al. [80] is obtained (cf. Figure 25b,d) when compared with those obtained using the original JC model (cf. Figure 25a,c). The modified JC model has an R-value of 0.991 compared with 0.962 for the original JC model. In addition, it has an AARE of 5.37% compared with 9.41% for the original JC model.

#### 3.2.13. Song JC-Based Modification

Song et al. [81] modified the original JC model to predict the flow behavior of titanium matrix composite under hot deformation. The softening term is modified to take the strain rate and temperature effect into account. The modified model can be expressed as

(39) $$\sigma =\left(A+B_{1}\varepsilon +B_{2}\varepsilon^{2}\right)\left(1+C\ln\varepsilon^{\cdot *}\right)e^{\left(\lambda_{1}\;T^{*}+\lambda_{2}T^{*}{}^{2}\;\right)\ln\varepsilon^{\cdot *}}$$

where $A,\;B_{1},\;B_{2},\;C,\;\lambda_{1}$, and $\lambda_{2}$ are material constants. Constants $A,\;B_{1},\;B_{2}$, and C can be determined in the same way that was explained in Section 3.2.5. At different strain rate values, Equation (39) can be expressed as

(40) $$\frac{\sigma }{\left(A+B_{1}\varepsilon +B_{2}\varepsilon^{2}\right)\left(1+C\ln\varepsilon^{\cdot *}\right)}=e^{\left(\lambda_{1}\;T^{*}+\lambda_{2}T^{*}{}^{2}\;\right)\ln\varepsilon^{\cdot *}}$$

Taking logarithms for both sides, after performing some rearrangements, Equation (40) can be introduced as

(41) $$\ln\left[\frac{\sigma }{\left(A+B_{1}\varepsilon +B_{2}\varepsilon^{2}\right)\left(1+C\ln\varepsilon^{\cdot *}\right)}\right]/\ln\varepsilon^{\cdot *}=\lambda_{1}\;T^{*}+\lambda_{2}T^{*}{}^{2}$$

**Figure 26:**
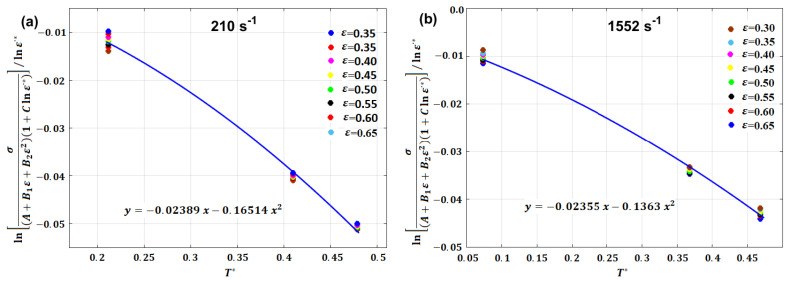
Determination of constants $\lambda_{1}$ and $\lambda_{2}$ at (**a**) 210 s^−1^ and (**b**) 1252 s^−1^.

By plotting the left side vs. $T^{*}$, different values for $\lambda_{1}$ and $\lambda_{2}$ can be obtained at different strain rate values, for which the average of each one can be obtained (cf. Figure 26).

**Figure 27:**
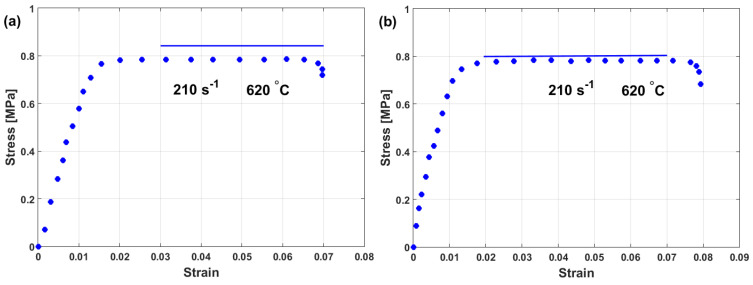
Experimental stresses (markers) compared to predicted stresses (solid lines) of titanium matrix composite under hot deformation using (**a**) the original JC and (**b**) the modified JC that was presented by Song et al. [81].

The modified JC model that was introduced by Song et al. [81] provided a good agreement between predicted stresses and experimental stresses when compared with those obtained using the original JC model (cf. Figure 27).

#### 3.2.14. Wang (2) JC-Based Modification

**Figure 28:**
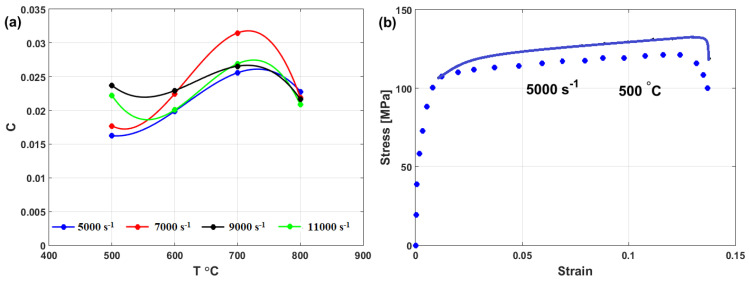
(**a**) Determination of C as a sine wave function and (**b**) comparison between experimental stresses (markers) and predicted stresses (solid line) obtained by the modified JC model that was presented Wang et al. [48].

Wang et al. [48] modified the strain rate term in the original JC model to predict the flow behavior of Inconel 718 at high strain rates and high temperatures. In this modification, the strain rate has been found to be correlated with temperature in a sine wave function. The modified JC model that was presented by Wang et al. [48] can be written as

(42) $$\sigma =\left(A+B\varepsilon^{n}\right)\left(1+C\left(\varepsilon^{\cdot },T\right)\ln\varepsilon^{\cdot *}\right)\left(1-T^{*}{}^{m}\right)$$

where A, B, n, and m are material constants that are determined in the same way that was explained for the original JC model (see Section 3.1). Constant C for Inconel 718 is represented by the sine wave function (cf. Figure 28a) as

$$C\left(\varepsilon^{\cdot },T\right)=0.0232\left(0.00372+0.0021\;sin\left(\frac{\varepsilon^{\cdot }-5000}{3000}\pi \right)\right)sin\left(\frac{T-500}{150}\pi \right)$$

Good agreement between experimental stresses and predicted stresses using the modified model for the flow behavior of Inconel 718 is thus obtained. However, this method does not provide accurate or precise predictions (Figure 28b).

Another sine wave approximation of constant $C\left(\varepsilon^{\cdot },T\right)$ was introduced by Xu et al. [88] to predict the flow behavior of SnSbCu alloy at a wide range of strain rates and different temperatures, with good predictions, in which $C\left(\varepsilon^{\cdot },T\right)$ is determined as

$$C\left(\varepsilon^{\cdot },T\right)=0.06-0.0232\left(0.05554+0.01777\;sin\left(\frac{\varepsilon^{\cdot }-1000}{5000}\pi \right)\right)sin\left(\frac{T-20}{300}\pi \right)$$

#### 3.2.15. Tan JC-Based Modification

**Figure 29:**
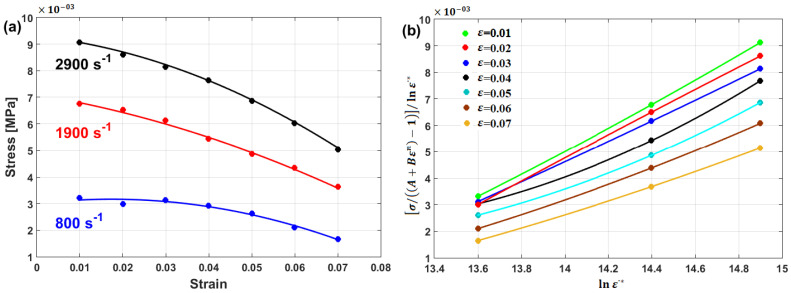
Determination of $C\left(\varepsilon ,\varepsilon^{\cdot }\right)$ (**a**) C vs. ε and (**b**) C vs. $\varepsilon^{\cdot }$.

Tan et al. [53] modified the JC model to predict the flow behavior of 7050-T7451 aluminum alloy under dynamic loading. Both strain hardening and softening are kept constant, as in the original JC model, while the strain rate term is modified to take the coupling effect of the strain and strain rate into account. The modified JC model that was introduced by Tan et al. [53] can be written as

(43) $$\sigma =\left(A+B\varepsilon^{n}\right)\left(1+C\left(\varepsilon ,\varepsilon^{\cdot }\right)\ln\varepsilon^{\cdot *}\right)\left(1-T^{*}{}^{m}\right)$$

where A, B, n,  and m are material constants that are determined in the same way that was explained for the original JC model (see Section 3.1). The constant, $C\left(\varepsilon ,\varepsilon^{\cdot }\right)$, is obtained by plotting $C=\left[\sigma /\left(\left(A+B\varepsilon^{n}\right)-1\right)\right]/\ln\varepsilon^{\cdot *}$ vs. both ε and $\varepsilon^{\cdot }$ (cf. Figure 29) in the original JC model at reference temperature and using polynomial fitting.

Constant $C\left(\varepsilon ,\varepsilon^{\cdot }\right)$ is determined by Tan et al. [53] as:

(44) $$C\left(\varepsilon ,\varepsilon^{\cdot }\right)=C_{0}+C_{1}\varepsilon +C_{2}\varepsilon^{2}+C_{3}\ln\varepsilon^{\cdot *}+C_{4}{\left(\ln\varepsilon^{\cdot *}\;\right)}^{2}+C_{5}\varepsilon \ln\varepsilon^{\cdot *}$$

where constants $C_{0},\;C_{1},\;C_{2},\;C_{3},\;C_{4},$ and $C_{5}$ are determined using regression analysis.

**Figure 30:**
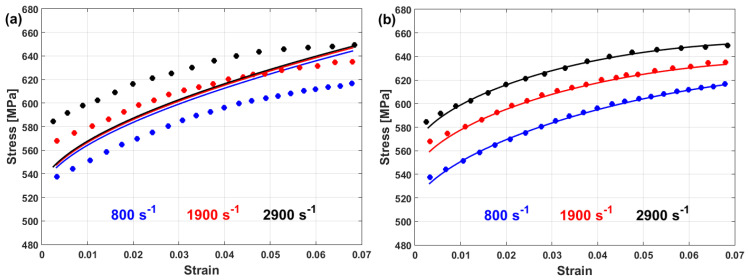
Experimental stresses (markers) compared to predicted stresses (solid lines) for 7050-T7451 aluminum alloy using (**a**) the original JC and (**b**) the modified JC model that was presented by Tan et al. [53].

Compared to the predictions of the original JC model (cf. Figure 30a), the modified JC model that was presented by Tan et al. [53] gave very good predictions for the flow behavior of the studied alloy at elevated temperatures (cf. Figure 30b). The good prediction of the flow stress using the modified model might be due to taking the interaction between the strain and strain rate into account.

#### 3.2.16. Chen JC-Based Modification

Chen et al. [82] modified the original JC model to predict the flow behavior of 7050-T745 aluminum alloy at high strain rates and different temperatures. The coupling effect of strain, strain rate, and temperature is taken into account in this modification. The modified JC model that was introduced by Chen et al. [82] can be expressed as

(45) $$\sigma =\left[\left(A+B\varepsilon^{n}\right)\left(\frac{1-{\left(\;\varepsilon^{\cdot }/\varepsilon^{\cdot }{}_{max}\right)}^{p2}*\tan h\left(\varepsilon \right)}{\exp\left(\varepsilon^{p1}\right)}\right){\left(\frac{T_{m}}{T}\right)}^{p3}\right]\;\;\;\;\;\;\;\;\;\;\;\;\;\;\;\;\;\;\;\;\;\;\;\;\;\;\;\;\;\;\;\;\;\;\;\;\;\;\;\;\;\;\;\;\;\times \left[1+C\ln\varepsilon^{\cdot *}\right]\left[1-{\left(1-\frac{\ln\varepsilon^{\cdot }{}_{max}-\ln\varepsilon^{\cdot }}{\ln\varepsilon^{\cdot }{}_{max}-\ln\varepsilon^{\cdot }{}_{min}}\right)}^{q}{\left(T^{*}\right)}^{m}\right]$$

Material constants A, B, n, C, m, p1, p2, p3, and q are determined using a generic algorithm developed by Chen et al. [184] to minimize the mean square error between experimental stresses and predicted stresses.

**Figure 31:**
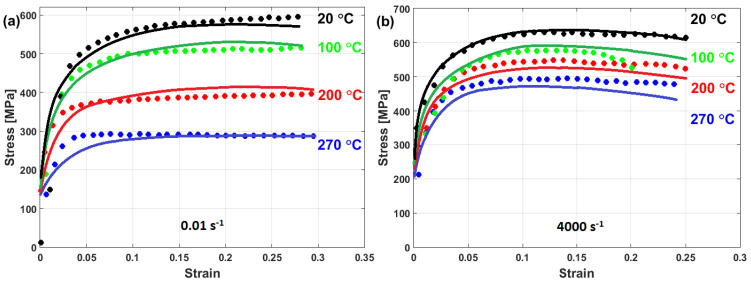
Experimental stresses (markers) compared to predicted stresses (solid lines) using a modified JC model that was presented by Chen et al. [82] for 7050-T745 aluminum alloy at high strain rates and different temperatures (**a**) 0.01 s^−1^ and (**b**) 4000 s^−1^.

The predicted stresses obtained using the modified JC model that was presented by Chen et al. [82] were found to be in good agreement with experimental stresses for 7050-T745 aluminum alloy at high strain rates and different temperatures for the tested alloy with RE less than 5%, which might be attributed to the interrelation between the hot working parameters (cf. Figure 31).

#### 3.2.17. Wang (3) JC-Based Modification

Wang et al. [83] modified the JC model to predict the flow behavior of a nickel-based superalloy at high strain rates and different temperatures. The coupling effect of anomalous temperature and strain rate dependences on flow stress is taken into account in this modification, using the strain rate term that was first presented by Rule and Jones [68] and modifying the softening term. The modified JC model that was introduced by Wang et al. [83] can be expressed as

(46) $$\sigma =\left(A+B\varepsilon^{n}\right)\left[1+C_{3}\ln\varepsilon^{\cdot *}+C_{4}\left(\frac{1}{C_{5}-\ln\varepsilon^{\cdot }}-\frac{1}{C_{5}}\right)\right]\;\;\;\;\;\;\;\;\;\;\;\;\;\;\;\;\;\;\;\;\;\;\;\;\;\;\;\;\;\;\;\;\;\;\;\;\;\times \left[1-{\left(T^{*}\right)}^{m}+D\;exp\left[-\frac{{\left(T-T_{p}\right)}^{2}}{2d^{2}}\right]\right]$$

Material constants $A,\;B,\;n,\;C_{3},\;C_{4},\;C_{5},m,D,d\;$, and $T_{p}$ are determined by creating a generic algorithm that minimizes the mean square error between experimental stresses and predicted stresses, as reported by Rule and Jones [68].

**Figure 32:**
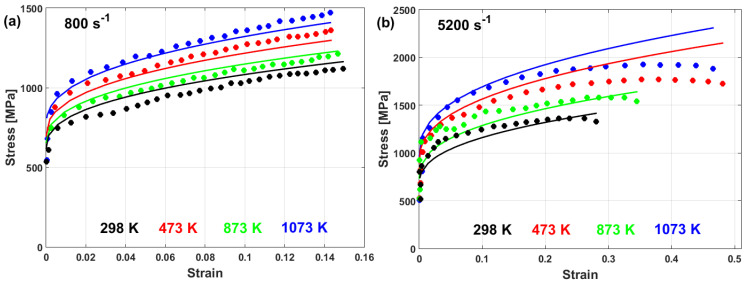
Comparison between experimental stresses (markers) and predicted stresses for GH4133B using the modified JC model that was presented by Wang et al. [83] at (**a**) 800 s^−1^ and (**b**) 5200 s^−1^.

The modified JC model that was introduced by Wang et al. [83] provided good predictions for the flow behavior of the tested alloy when predicted stresses were compared to experimental stresses (cf. Figure 32).

#### 3.2.18. Shokry (1) JC-Based Modification

Shokry [84] introduced a modification of the JC model for the prediction of the flow behavior of alloy 800H at elevated temperatures and intermediate strain rates. The modification takes the coupling effect between strain and both strain rate and temperature into account. The modified JC that was presented by Shokry [84] can be expressed as

(47) $$\sigma =\left(A+B_{1}\varepsilon +B_{2}\varepsilon^{2}+B_{3}\varepsilon^{3}\right)\left(1+\left(C_{1+}C_{2}\varepsilon \right)\ln\varepsilon^{\cdot *}\right)\left(1-T^{*}{}^{\left(m_{1+}m_{2}\varepsilon \right)}\right)$$

where constants $A,\;B_{1},\;B_{2}$, and $B_{3}$ are determined by fitting the experimental data of stress and strain at the reference strain rate and reference temperature with three-order polynomial functions (see Section 3.2.5). Shokry [84] determined both strain rate constants $C_{1}$ and $C_{2}$ and both softening constants $m_{1}$ and $m_{2}$ at once using the Kalman filter technique, a mathematical method for the determination of constants that minimizes the mean square error between experimental and predicted stresses [185].

**Figure 33:**
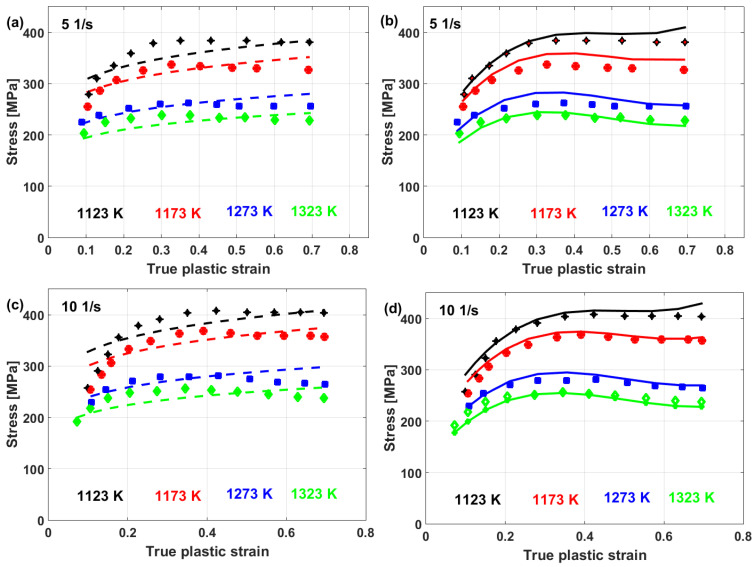
Comparison between experimental stresses (markers) and predicted stresses of alloy 800H obtained using the original JC model (dashed lines) and the modified JC model that was presented by Shokry [84] (solid lines) at (**a**,**b**) 5 s^−1^ and (**c**,**d**) 10 s^−1^.

A comparison between experimental stresses and predicted stresses obtained by the original JC and the modified JC that was presented by Shokry [84] is shown in Figure 33. The figure shows that the modified model can predict the flow behavior of the tested alloy better than the original JC model, with an R-value of 0.98 compared with 0.91 for the original JC model. The modified JC model that was presented by Shokry [84] provided good predictions of the flow behavior of a powder metallurgy nanoquasicrystalline Al9_3_Fe_3_Cr_2_Ti_2_ alloy at high temperatures and different strain rates [61] when compared with other models, with R and AARE values of 0.98 and 7.8%, respectively, compared with 0.92 and 12.7% for the original JC model, respectively.

#### 3.2.19. Zhao JC-Based Modification

Zhao et al. [85] used the modification of the original JC that was introduced by Lin et al. [73] and established a sine wave function for the strain rate term, as performed in [48], to predict the dynamic flow behavior of FeCr alloy manufactured using laser-additive manufacturing. The strain-hardening term is also modified by fitting stress with strain at the reference conditions, with three-order polynomial functions instead of two-order polynomial functions. The modified JC model that was introduced by Zhao et al. [85] can be written as

(48) $$\sigma =\left(A+B_{1}\varepsilon +B_{2}\varepsilon^{2}+B_{3}\varepsilon^{3}\right)\left(1+C_{1}\left(\varepsilon^{\cdot },T\right)\ln\varepsilon^{\cdot *}\right)\exp\left[\left(\lambda_{1}+\lambda_{2}\ln\varepsilon^{\cdot *}\right)\left(T-T_{r}\right)\right]$$

where $A,\;B_{1},\;B_{2},B_{3},\;\lambda_{1},$ and $\lambda_{2}$ are material constants that are determined in the same way that was explained for the modified JC model that was introduced by Lin et al. [48] (see Section 3.2.5). Constant C for the FeCr alloy is represented by

$$C\left(\varepsilon^{\cdot },T\right)=\left(0.0031+0.0273\;sin\left(\frac{\varepsilon^{\cdot }-900}{800}\pi \right)\right)sin\left(\frac{T-100}{800}\pi \right)$$

**Figure 34:**
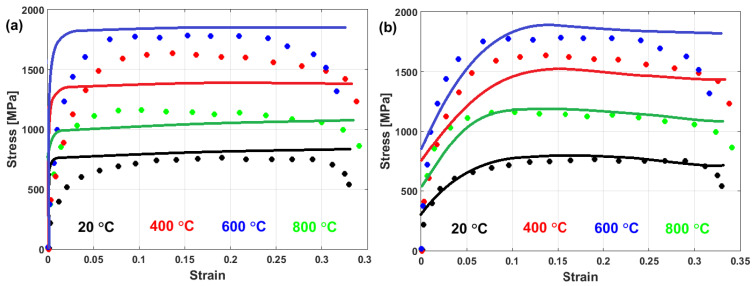
Comparison between experimental stresses (solid lines) and predicted stresses (solid lines) for FeCr alloy at 8000 s^−1^ obtained by (**a**) the original JC model and (**b**) the modified JC model that was presented by Zhao et al. [85].

Good agreement between experimental stresses and predicted stresses is obtained using the modified JC model that was introduced by Zhao et al. [85], with R: 0.87–0.99 and AARE: 7.6–22.7% when compared with those obtained using the original JC model with R: 0.83–0.88 and AARE: 21.8–44.8%. However, very accurate predictions were not found (cf. Figure 34).

#### 3.2.20. Iturbe JC-Based Modification

**Figure 35:**
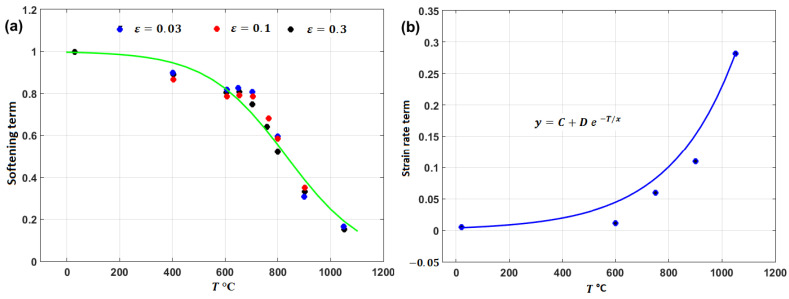
Exponential fitting of temperature with (**a**) the thermal softening term and (**b**) the strain hardening term.

Iturbe et al. [49] introduced a modification of the JC model to predict the flow behavior of Inconel 718 nickel superalloy under hot deformations. The Lurdos model [186] is used for the strain-hardening term, and the coupling effect between the temperature and strain rate is taken into account. The modified JC model that was introduced by Iturbe et al. [49] can be written as

(49) $$\sigma =\left[\sigma_{s}+\left(\sigma_{0}-\sigma_{s}+A\varepsilon^{n}\exp\left(-r\varepsilon \right)\right)\right]\left[\frac{1}{1+e^{-m\left(T-B\right)}}\right]\left[1+\left(C+De^{-T/x}\right)\ln\left(\varepsilon^{\cdot *}\right)\right]$$

where $\sigma_{s}$ is saturation stress, and $\sigma_{0}$ is yield stress. A, n, r,m,B,C,D,  and x are material constants. At the reference strain rate and reference temperature, constants A, n, and r are determined by fitting the experimental stress and strain data. At the reference temperature, constants C,D, and x are determined using exponential fitting between the softening term in the original JC model with T (cf. Figure 35a), and exponential fitting between the strain rate term and T is used to compute constants m and B at the reference strain rate (cf. Figure 35b).

**Figure 36:**
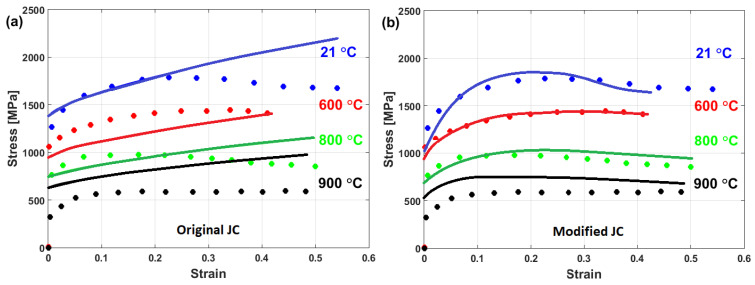
Comparison between experimental stresses (solid lines) and predicted stresses (dashed lines) for Inconel 718 superalloy obtained by (**a**) the original JC model and (**b**) the modified JC model that was introduced by Iturbe et al. [49].

The modified JC model that was presented by Iturbe et al. [49] provided good predictions of the flow behavior of the tested alloy compared with the predicted stresses obtained using the original JC model for the tested alloy. Still, precise predictions were not achieved (cf. Figure 36).

#### 3.2.21. Tao JC-Based Modification

**Figure 37:**
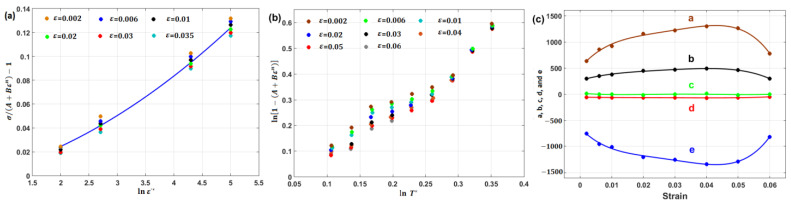
Determination of constants (**a**) $C_{1}$ and $C_{2}$; (**b**) different values of a, b, c, d, and d at different strains; (**c**) fitting a, b, c, d, and d as functions of strain.

Tao et al. [63] presented a modification of the original JC model for the prediction of the flow behavior of a Ti-6Al-4V tube during warm bending at different strain rate and temperature ranges. In their modification, the strain rate term of the original JC model is replaced by a strain rate term that was previously introduced by Kang et al. [69], while the softening term is modified considering constant m in the original JC model as a function of temperature. The modified JC that was presented by Tao et al. [63] can be expressed as

(50) $$\sigma =\left(A+B\varepsilon^{n}\right)\left(1+C_{1}\ln\left(\varepsilon^{\cdot *}\right)+C_{2}{\left(\ln\left(\varepsilon^{\cdot *}\right)\right)}^{2}\right)\left(1-T^{*a+bT^{*}+cT^{*}{}^{2}+dT^{*}{}^{3}+eT^{*}{}^{4}}\right)$$

where $A,B,\;n,\;C_{1},C_{2},a,b,c,d,$ and e are material constants. Constants A,B, and n are determined as explained for the original JC model in Section 3.1. Constants $C_{1}$ and $C_{2}$ are determined using quadratic fitting by plotting $\sigma /\left(A+B\varepsilon^{n}\right)-1$ vs. $\ln\left(\varepsilon^{\cdot *}\right)$ at the reference temperature (cf. Figure 37a). Thus, the parameters a,b,c,d, and d are determined to have different values at different strain values when plotted for $\ln\left[1-\left(+B\varepsilon^{n}\right)\right]\;\mathrm{vs}.\;\ln\;\left(T^{*}\right)$ (cf. Figure 37b), and, finally, they are expressed as fourth-order polynomials fitted by plotting the obtained values vs. strain (cf. Figure 37c).

**Figure 38:**
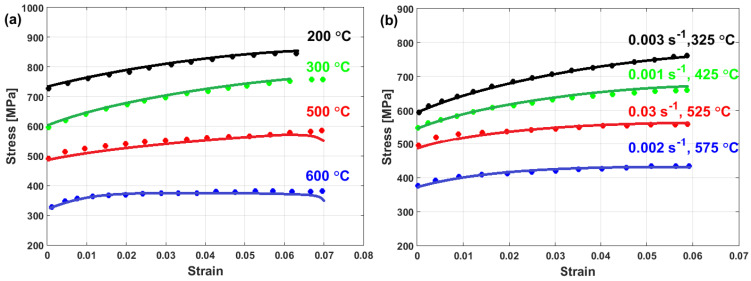
Experimental stresses (markers) compared to predicted stresses (solid lines) using the modified JC model that was presented by Tao et al. [63] for Ti-6Al-4V alloy for (**a**) the tested range of temperature and strain rates and (**b**) new experiments with different strain rates and temperatures for verification.

Good agreement can be seen between the experimental stresses and predicted stresses for the prediction of the flow behavior of the Ti-6Al-4V alloy obtained using the modified JC model that was introduced by Tao et al. [63] (cf. Figure 38a). The modified JC model is verified by using new experimental data that were not included in the determination of the constants. Very good predictions are also obtained, which enhances the use of the coupling effect of strain and temperature in this modification (cf. Figure 38b).

#### 3.2.22. He JC-Based Modification

He et al. [86] introduced a modification of the original JC model to predict the flow behavior of a 10%Cr steel alloy at elevated temperatures and different strain rates. In their modification, strain-hardening terms are modified, and the coupling effect between the strain and strain rate is presented. The modified JC model that was introduced by He et al. [86] can be written as

(51) $$\sigma =A_{1}\varepsilon^{n_{1}}.\left(1+\left(b_{1}+b_{2}\varepsilon +b_{3}\varepsilon^{2}\right)\ln\left(\varepsilon^{\cdot *}\right)\right)\exp\left[\left(\lambda_{1}+\lambda_{2}\varepsilon \right)T^{*}\right]$$

where $A_{1},\;n_{1},\;b_{1},b_{2},b_{3},\lambda_{1}$, and $\lambda_{2}$ are material constants. At the reference strain rate and reference temperature, Equation (51) reduces to $\sigma =A_{1}\varepsilon^{n_{1}}$; by taking logarithms for both sides and plotting lnσ vs. lnε, constant $A_{1}$ can be determined from the intercept, while constant $n_{1}$ can be determined from the slope. At the reference temperature, Equation (51) lowers to:

(52) $$\sigma =A_{1}\varepsilon^{n_{1}}.\left(1+\left(b_{1}+b_{2}\varepsilon +b_{3}\varepsilon^{2}\right)\ln\left(\varepsilon^{\cdot *}\right)\right)$$

**Figure 39:**
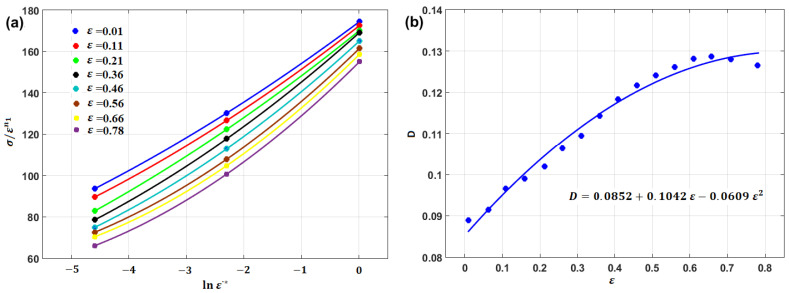
Determination of coefficients $b_{1},b_{2}$, and $b_{3}$: (**a**) determination of different values of D at different ε values and (**b**) quadratic fitting between D and ε.

To obtain constants $b_{1},b_{2}$, and $b_{3}$, a new parameter, D, is introduced, in which $D=b_{1}+b_{2}\varepsilon +b_{3}\varepsilon^{2}$; then, after performing some rearrangements, Equation (51) can be written as $\sigma /\varepsilon^{n_{1}}=A_{1}+A_{1}D\ln\left(\varepsilon^{\cdot *}\right)$; after that, by plotting $\sigma /\varepsilon^{n_{1}}$ vs. $\ln\left(\varepsilon^{\cdot *}\right)$, different values can be obtained from the slope $\left(A_{1}D\right)$ at different strain values (cf. Figure 39a). Finally, the obtained values of D can be plotted vs. its corresponding ε values, and quadratic fitting can be implemented to obtain the values of $b_{1},b_{2}$, and $b_{3}$ (cf. Figure 39b). Constants $\lambda_{1}\;$ and $\lambda_{2}$ can be determined in the same way that was explained in Section 3.2.5.

**Figure 40:**
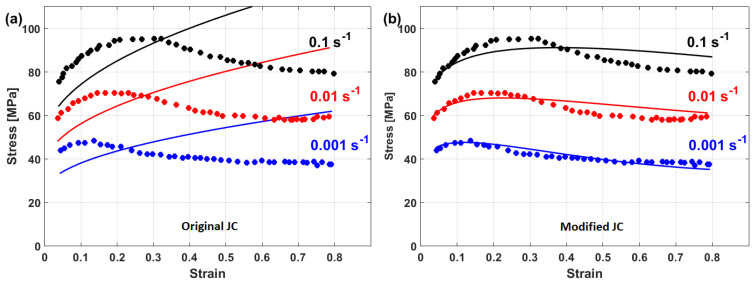
Experimental stresses (solid lines) compared to predicted stresses (markers) for 10%Cr steel alloy under hot deformation using (**a**) the original JC model and (**b**) the modified JC model that was presented by He et al. [86].

Comparisons between the experimental stresses and predicted stresses for the 10%Cr steel alloy obtained using both the JC model and the modified JC model that was introduced by He et al. [86] are shown in Figure 40a and 40b, respectively. The modified JC model achieved a better agreement with the experimental stresses than the original JC model; however, not the predictions were not very precise.

#### 3.2.23. Hou X. JC-Based Modification

Hou X. et al. [87] modified the original JC model for the prediction of the flow behavior of Ti-6Al-4V at elevated temperatures and different strain rates. The strain-hardening term is correlated with temperature in their modification, while both the strain rate and softening terms are kept as in the original JC model. The modified JC model that was presented by Hou X. et al. [87] can be written as

(53) σ=A+B1+m1lnTTrεn1+Clnε·*1−T*m2

where $A,\;B,\;m_{1},n,C$, and $m_{2}$ are material constants. At the reference strain rate, after introducing a new parameter, Q=B1+m1lnTTr, considering A is the yield stress and $m_{2}=m,$ which are determined based on the original JC model, Equation (53) reduces to

(54) $$\sigma =\left(A+Q\varepsilon^{n}\right)\left(1-T^{*}{}^{m}\right)$$

After performing some rearrangements, Equation (54) can be written as

(55) $$\frac{\sigma }{1-T^{*}{}^{m}}-A=Q\varepsilon^{n}$$

Taking logarithms for both sides and plotting $\ln\left[\sigma /\left(1-T^{*}{}^{m}\right)-A\right]$ vs. lnε, constant n is the slope, and Q can be determined from the intercept. Different values of Q are obtained at different values of T; then, constants B and $m_{1}$ can be determined by fitting the obtained data of Q with $T/T_{r}$. Constants C and $m_{2}$ can be determined in the same way that was explained for the original JC model (see Section 3.1).

**Figure 41:**
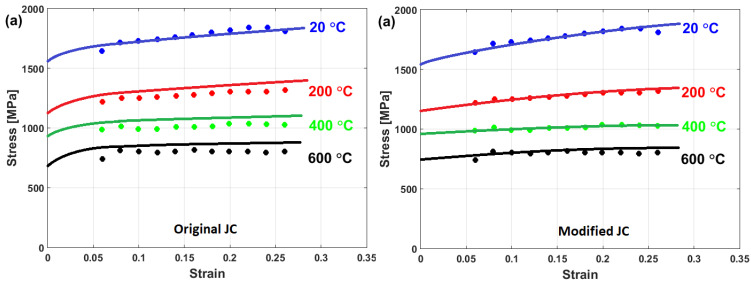
Experimental stresses (markers) compared to predicted stresses (solid lines) for Ti-6Al-4V alloy under hot deformation using (**a**) the original JC model and (**b**) the modified JC model that was presented by Hou X. et al. [87].

A comparison between the experimental stresses and predicted stresses for the Ti-6Al-4V alloy under hot deformation using both the original JC model (cf. Figure 41a) and the modified JC model that was presented by Hou X. et al. [87] (cf. Figure 41b) is shown in Figure 41. As can be seen, the modified JC model gave a more accurate prediction (RE = 4.19%) than the original JC model (RE = 10.43%), which might be a result for taking the interaction of strain and temperature into account.

Promoppatum et al. [187] studied the effect of constitutive models for the prediction of the mechanical behavior of the laser powder bed fusion of Ti-6Al-4V alloy using finite element simulations and the modified JC model that was introduced by Hou X. et al. [87], along with other models. The results showed that temperature-dependent strain hardening has a minimal effect on the predicted stress and strain fields. On the other hand, the rate-dependent term has a large effect on them. In contrary findings, Kugalur-Palanisamy et al. [188] reported the significance of the temperature-dependent strain hardening consequence on the deformation behavior of Ti-6Al-4V alloy through the machining process when using the modified JC model that was introduced by Hou X. et al. [87], along with others, in finite element simulations of the orthogonal cutting process.

#### 3.2.24. Zhang JC-Based Modification

Zhang et al. [89] modified the softening term in the original JC to predict the flow behavior of AZ31 magnesium alloy at high temperatures and high strain rates. The modification is based on the dependence of the softening parameter on temperature, which can be expressed as

(56) $$\sigma =\left(A+B\varepsilon^{n}\right)\left(1+C\ln\left(\varepsilon^{\cdot *}\right)\right)\left(1-T^{*}{}^{m\left(T\right)}\;\right)$$

where A,B, n, and C are material constants that are determined in the same way as explained in Section 3.1. In their modification, $m\left(T\right)$ is determined as $e^{m_{0}+m_{1}T+m_{2}T^{2}}$. After performing some rearrangements, at different values of strain rate, Equation (56) can be written as

(57) $$1-\frac{\sigma }{\left(A+B\varepsilon^{n}\right)\left(1+C\ln\left(\varepsilon^{\cdot *}\right)\right)}=T^{*}{}^{m\left(T\right)}$$

**Figure 42:**
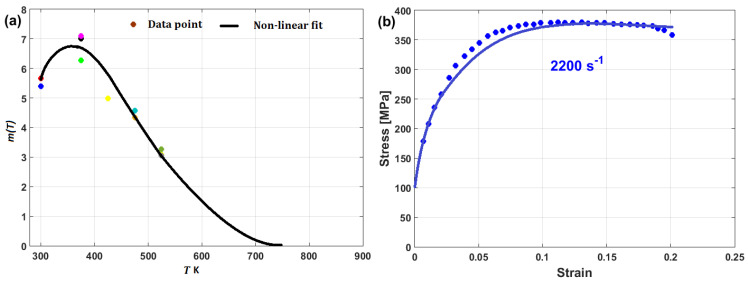
(**a**) Quadratic polynomial of $m\left(T\right)$ and (**b**) comparison between experimental stresses (markers) and predicted stresses (solid line) for AZ31 magnesium alloy obtained by the modified JC model that was introduced by Zhang et al. [89].

By taking logarithms for both sides, $\ln\left[1-\sigma \left(A+B\varepsilon^{n}\right)\left(1+C\ln\left(\varepsilon^{\cdot *}\right)\right)\right]$ can be plotted vs. $\ln T^{*}$, in which $m\left(T\right)$ is the slope of the equation. At different temperature and strain rate values, different values for $m\left(T\right)$ can be obtained. Constants $m_{0},m_{1}$, and $m_{2}$ are obtained by quadratically fitting $m\left(T\right)$ vs. T using those values (cf. Figure 42a).

A comparison between experimental stresses and predicted stresses obtained using the modified JC model that was introduced by Zhang et al. [89] for the prediction of the flow behavior of AZ31 magnesium alloy is shown in Figure 42b. Good agreement is obtained, which might be attributed to the dependence of the softening parameter on temperature.

#### 3.2.25. Niu JC-Based Modification

Niu et al. [90] introduced a modification of the original JC model for the accurate prediction of the flow behavior of A356 alloy at elevated temperatures and different strain rates. In their modification, the strain-hardening rate is modified to be interconnected with strain. Furthermore, the strain rate parameter is established to be interrelated with the strain and strain rate. In addition, the softening parameter is considered a function of the strain, strain rate, and temperature. The modified JC model that was presented by Niu et al. [90] can be written as

(58) $$\sigma =\left(A\varepsilon^{B+C\varepsilon +D/\varepsilon }\right)\left(1+E\ln\varepsilon^{\cdot *}\right)\exp\left[F\left(T-T_{r}\right)\right]$$

where A, B, C, and A are material constants. Constant E is a function of ε and $\varepsilon^{\cdot }$, and constant F is a function of ε, $\varepsilon^{\cdot }$, and $\left(T-T_{r}\right)$. Constants E and F can be expressed as

(59) $$E=E_{0}+E_{1}\varepsilon +E_{2}\varepsilon^{2}+E_{3}\varepsilon^{\cdot }+E_{4}\varepsilon^{\cdot }{}^{2}+E_{5}\varepsilon \varepsilon^{\cdot }$$

(60) $$F=F_{0}+F_{1}\varepsilon +F_{2}\varepsilon^{\cdot }+F_{3}\left(T-T_{r}\right)+F_{4}\varepsilon^{3}+F_{5}\varepsilon^{\cdot }{}^{3}+F_{6}{\left(T-T_{r}\right)}^{3}\;+F_{7}\varepsilon \varepsilon^{\cdot }{\left(T-T_{r}\right)}^{3}$$

At the reference strain rate and reference temperature, Equation (58) lowers to

(61) $$\sigma =A\varepsilon^{B+C\varepsilon +D/\varepsilon }$$

By plotting σ vs. ε, constants A, B, C, and D can be obtained using regression analysis. After performing some rearrangements, at the reference temperature, Equation (58) reduces to

(62) $$\frac{\sigma }{A\varepsilon^{B+C\varepsilon +D/\varepsilon }}-1=E\ln\varepsilon^{\cdot *}$$

Consequently, constants $E_{0},E_{1},E_{2},E_{3},E_{4}$, and $E_{5}$ can be determined using regression analysis. At different values of strain rates, and taking logarithms after performing some rearrangements, Equation (58) can be written as

(63) $$\ln\left[\frac{\sigma }{\left(A\varepsilon^{B+C\varepsilon +D/\varepsilon }\right)\left(1+E\ln\varepsilon^{\cdot *}\right)}\right]=F\left(T-T_{r}\right)$$

Accordingly, constants $F_{0},F_{1},F_{2},F_{3},F_{4},F_{5},F_{6}$, and $F_{7}$ can be determined using regression analysis.

**Figure 43:**
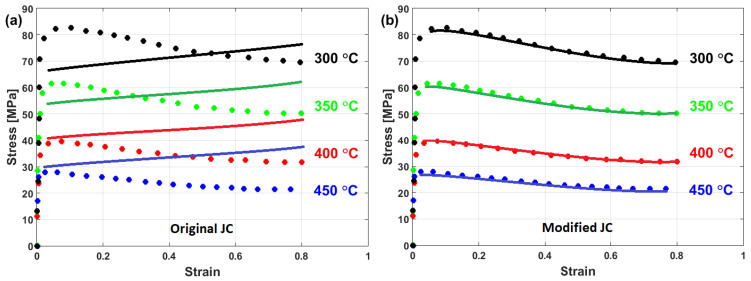
Comparison between experimental stresses (markers) and predicted stresses (solid lines) for A356 alloy obtained by (**a**) original JC and (**b**) modified JC that was presented by Niu et al. [90].

Very accurate predictions of the flow stress for the tested alloy are obtained when comparing predicted stresses obtained using the modified JC model that was introduced by Niu et al. [90], with experimental stresses with AARE = 1.46% (cf. Figure 43b). On the other hand, the original JC model provided inaccurate predictions, with AARE = 26.31% (cf. Figure 43a). The coupling effect of strain, strain rate, and temperature might be a reason for the accurate predictions using the modified JC model.

#### 3.2.26. Chakrabarty JC-Based Modification

Chakrabarty et al. [91] presented a modification of the original JC model to predict the flow behavior of copper at very high strain rates. The modified JC model that was presented by Chakrabarty et al. [91] can be expressed as

(64) $$\sigma =\left(A+B\varepsilon^{n}\right)\left[1+C\ln\frac{\varepsilon^{\cdot }}{\varepsilon_{0}^{.}}{\left(\frac{\varepsilon^{\cdot }}{\varepsilon_{c}^{.}}\right)}^{D}\right]\left(1-T^{*}{}^{m}\;\right)$$

where constants A, B,n, and m are determined in the same way that was explained for the original JC model (see Section 3.1). At the reference temperature, after performing some rearrangements, Equation (64) can be expressed as

(65) $$\frac{\sigma }{A+B\varepsilon^{n}}=1+C\ln\frac{\varepsilon^{\cdot }}{\varepsilon_{0}^{.}}{\left(\frac{\varepsilon^{\cdot }}{\varepsilon_{c}^{.}}\right)}^{D}$$

**Figure 44:**
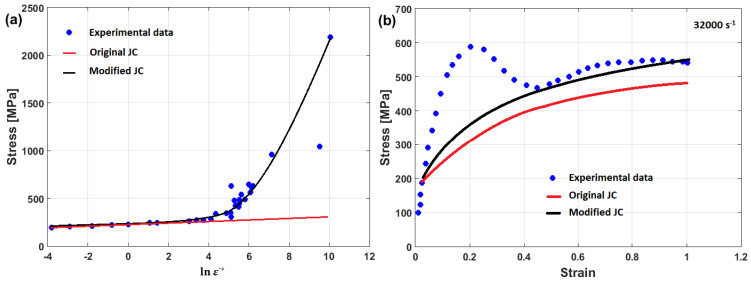
Comparison of experimental stresses (markers) and predicted stresses (solid lines) by using the JC model and the modified JC model that was presented by Chakrabarty et al. [91]: (**a**) stress vs. logarithmic strain rate and (**b**) stress vs. strain.

Chakrabarty et al. [91] determined both constants, $\varepsilon_{c}^{.}$ and D, using a fitting toolbox in MATLAB that is based on the non-linear least squares method (cf. Figure 44a). The modified JC model provided good predictions of the flow stress of copper when compared with the original JC model (cf. Figure 44b).

#### 3.2.27. Li JC-Based Modification

Li et al. [92] modified the JC model to predict the flow behavior of Roma Plastilina No. 1 (RP # 1) clay, which is used as a backing material in ballistic tests, as

(66) $$\sigma =\left(A_{1}\varepsilon_{p}^{n}-A_{2}e^{-K\varepsilon_{p}}\right)\left[1+C_{1}\ln\left(\varepsilon^{\cdot *}\right)\;\;\;\;\;\;\;\;\;\;\;\;\;\;\;\;\;\;\;\;\;\;\;\;\;\;\;\;\;\;\;\;\;\;\;\;\;\;\;\;\;\;\;\;\;\;\;\;\;\;\;\;\;\;\;\;\;\;\;\;\;\;\;\;\;\;\;\;\;\;\;\;\;\;\;\;\;\;\;\;\;\;\;\;\;\;\;\;\;\;\;\;\;\;\;\;\;\;\;\;\;\;\;\;\;+C_{2}{\left(\ln\left(\varepsilon^{\cdot *}\right)\right)}^{2}\right]\exp\left[m_{1}+m_{2}\ln\left(\varepsilon^{\cdot *}\right)+m_{3}{\left(\ln\left(\varepsilon^{\cdot *}\right)\right)}^{2}\right]\left(T-T_{r}\right)$$

where $A_{1},\;n,\;A_{2},K,C_{1},C_{2},m_{1},m_{2}$, and $m_{3}$ are material constants. At the reference strain rate and reference temperature, Equation (66) reduces to

(67) $$\sigma =\left(A_{1}\varepsilon_{p}^{n}-A_{2}e^{-K\varepsilon_{p}}\right)$$

**Figure 45:**
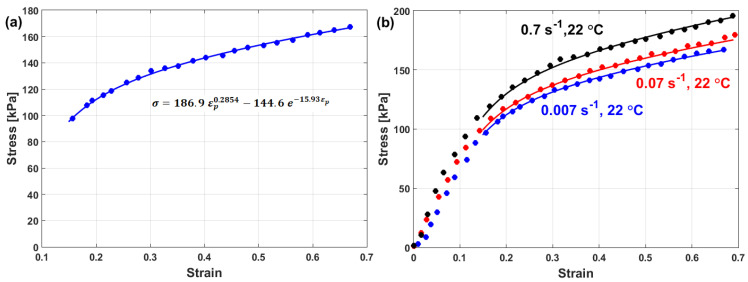
(**a**) Fitting true stress true strain to obtain constants $A_{1},\;n,\;A_{2}$, and K at reference values and (b) comparison between experimental stresses (markers) and predicted stresses (solid line) for ballistic Roma Plastilina NO. 1 clay obtained using the modified JC model that was introduced by Li et al. [92].

By plotting σ vs. ε, constants $A_{1},\;n,\;A_{2}$, and K can be determined using curve-fitting based on the non-linear least squares method incorporated in MATLAB (cf. Figure 45a). At the reference temperature, constants $C_{1}$ and $C_{2}$ can be determined using the same method that was explained in Section 3.2.21. Taking the logarithm, after performing some rearrangements, at different strain rate values, Equation (66) can be written as

(68) $$\ln\left[\frac{\sigma }{\left(A_{1}\varepsilon_{p}^{n}-A_{2}e^{-K\varepsilon_{p}}\right)\left[1+C_{1}\ln\left(\varepsilon^{\cdot *}\right)+C_{2}{\left(\ln\left(\varepsilon^{\cdot *}\right)\right)}^{2}\right]}\right]=m\left(\ln\left(\varepsilon^{\cdot *}\right)\right)\left(T-T_{r}\right)$$

where $m\left(\ln\left(\varepsilon^{\cdot *}\right)\right)$ is the slope of the equation. At different temperatures and different strain rates, different values of $m\left(\ln\left(\varepsilon^{\cdot *}\right)\right)$ can be obtained, in which constants $m_{1},m_{2}$, and $m_{3}$ can be obtained by quadratically fitting $m\left(\ln\left(\varepsilon^{\cdot *}\right)\right)$ vs. $\ln\left(\varepsilon^{\cdot *}\right)$ using those values.

The modified JC model that was introduced by Li et al. [92] succeeded in providing good predictions of the flow behavior of the tested alloy at different strain rates and different temperatures, with R values ranging from 0.9896 to 0.9995 and AARE values ranging from 15.74% to 28.93% (cf. Figure 45b).

#### 3.2.28. Qian JC-Based Modification

Qian et al. [93] presented a modification of the JC model for the prediction of a CuCrZr alloy at high strain rates and high temperatures. The coupling effect between the strain and both the strain rate and temperature is taken into consideration in this modification. The modified JC model that was presented by Qian et al. [93] can be expressed as

(69) $$\sigma =\left(A+B\varepsilon^{n}\right)\left(1+C\left(\varepsilon ,\ln\left(\varepsilon^{\cdot *}\right)\right)\ln\left(\varepsilon^{\cdot *}\right)\right)\left(1-m_{0}T^{*}{}^{m_{1}+m_{2}\varepsilon }\;\right)$$

where $A,\;B,\;n,m_{0},m_{1},$ and $m_{2}$ are material constants. Qian et al. [93] implemented the strain rate parameter $C\left(\varepsilon ,\ln\left(\varepsilon^{\cdot *}\right)\right)$, which was first presented by Abd El-Aty et al. [189] in their modification, which can be expressed as

(70) $$C\left(\varepsilon ,\ln\left(\varepsilon^{\cdot *}\right)\right)=C_{0}+C_{1}\varepsilon +C_{2}\varepsilon^{2}+C_{3}\varepsilon \ln\left(\varepsilon^{\cdot *}\right)+C_{4}\ln\left(\varepsilon^{\cdot *}\right)+C_{5}{\left(\ln\left(\varepsilon^{\cdot *}\right)\right)}^{2}$$

Constants A, B, and n are determined as explained in the original JC model (see Section 3.1). At the reference temperature, Equation (69) lowers to

(71) $$\sigma =\left(A+B\varepsilon^{n}\right)\left(1+C\left(\varepsilon ,\ln\left(\varepsilon^{\cdot *}\right)\right)\ln\left(\varepsilon^{\cdot *}\right)\right)$$

Constants $C_{0},C_{1},C_{2},C_{3},C_{4}$, and $C_{5}$ can be determined by plotting $\sigma /\left(A+B\varepsilon^{n}\right)-1\;\mathrm{vs}.\;\ln\left(\varepsilon^{\cdot *}\right)$ using regression analysis. Taking the logarithm after performing some rearrangements and at different strain rate values, Equation (69) can be expressed as

(72) $$\ln\left[1-\frac{\sigma }{\left(A+B\varepsilon^{n}\right)\left(1+C\left(\varepsilon ,\ln\left(\varepsilon^{\cdot *}\right)\right)\ln\left(\varepsilon^{\cdot *}\right)\right)}\right]=\ln m_{0}+\left(m_{1}+m_{2}\varepsilon \right)\ln T^{*}$$

Constant $m_{0}$ can be determined from the intercept of the equation, while $m_{1}$ and $m_{2}$ can be determined from the slope, as explained in Section 3.2.24.

**Figure 46:**
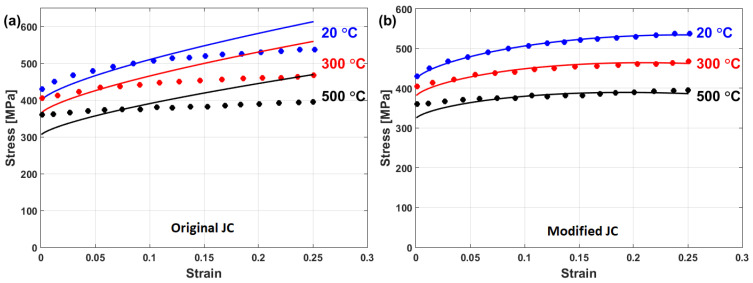
Comparison between experimental stresses (markers) and predicted stresses (solid lines) for CuCrZr alloy obtained by (**a**) the original JC model and (**b**) the modified JC model that was presented by Qian et al. [93].

The modified JC model that was introduced by Qian et al. [93] improved the accuracy of the prediction of the flow behavior of the CuCrZr alloy, with AARE values ranging from 0.59% to 3.22% (cf. Figure 46b) compared with the predictions obtained using the original JC model, with AARE values range from 7.41% to 10.22% (cf. Figure 46a). Taking the interconnection between the strain, strain rate, and temperature might be a reason for the obtained accuracy of the predictions.

#### 3.2.29. Liu JC-Based Modification

Liu et al. [94] modified both the strain-hardening and strain rate terms in the original JC model to predict the flow behavior of SWRH82B steel alloy at high temperatures and different strain rates. The modified JC that was presented by Liu et al. [94] can be expressed as

(73) $$\sigma =\left(\frac{A*\varepsilon }{B+\varepsilon }\right)\left(1+C\left(\varepsilon ,\ln\left(\varepsilon^{\cdot *}\right)\right)\ln\left(\varepsilon^{\cdot *}\right)\right)\left(1-T^{*m\;}\;\right)$$

where A, B, and m are material constants. Parameter $C\left(\varepsilon ,\ln\left(\varepsilon^{\cdot *}\right)\right)$ is determined as

(74) $$C\left(\varepsilon ,\ln\left(\varepsilon^{\cdot *}\right)\right)=\exp\left(C_{0}+C_{1}\varepsilon +C_{2}\varepsilon^{2}+C_{3}\varepsilon \ln\left(\varepsilon^{\cdot *}\right)+C_{4}\ln\left(\varepsilon^{\cdot *}\right)+C_{5}{\left(\ln\left(\varepsilon^{\cdot *}\right)\right)}^{2}\right)$$

At the reference strain rate and reference temperature, Equation (73) lowers to

(75) $$\sigma =\left(\frac{A*\varepsilon }{B+\varepsilon }\right)$$

By plotting σ vs. ε, constants A and B can be determined using regression analysis. Taking logarithms for both sides after performing some rearrangements and at the reference temperature, Equation (73) can be written as

(76) $$\ln\left[\frac{\sigma }{\left(\frac{A*\varepsilon }{B+\varepsilon }\right)}-1\right]-\ln\left(\varepsilon^{\cdot *}\right)\;\;\;\;\;\;\;\;\;\;\;\;\;\;\;\;\;\;\;\;\;\;\;\;\;\;\;\;\;\;\;\;=C_{0}+C_{1}\varepsilon +C_{2}\varepsilon^{2}+C_{3}\varepsilon \ln\left(\varepsilon^{\cdot *}\right)+C_{4}\ln\left(\varepsilon^{\cdot *}\right)+C_{5}{\left(\ln\left(\varepsilon^{\cdot *}\right)\right)}^{2}$$

where constants $C_{0},C_{1},C_{2},C_{3},C_{4}$, and $C_{5}$ can be determined using regression analysis.

**Figure 47:**
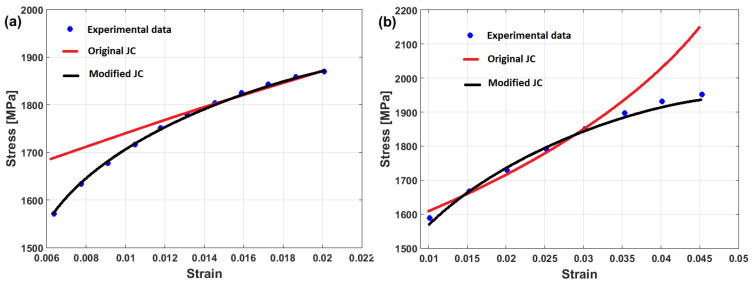
Experimental stresses (markers) compared to predicted stresses (solid lines) for SWRH82B steel alloy at a quasi-static strain rate and room temperature for circular wire with diameters (**a**) 4.2 mm and (**b**) 5.2 mm.

The modified JC model that was presented by Liu et al. [94] showed good predictability (cf. Figure 47b) when compared with the original JC model (cf. Figure 47a) for the prediction of the flow behavior of SWRH82B steel alloy with two different specimen configurations.

#### 3.2.30. Yu JC-Based Modification

Yu et al. [60] proposed a modification of the original JC model to predict the flow behavior of TA23 titanium alloy under hot deformation. In their modification, the softening term is modified to take the coupling effect between the strain rate and temperature into account. The modified JC model that was presented by Yu et al. [60] can be written as

(77) $$\sigma =\left(A+B_{1}\varepsilon +B_{2}\varepsilon^{2}\right)\left(1+C_{1}\ln\varepsilon^{\cdot *}\right)\exp\left[\left(\lambda_{1}+\lambda_{2}\ln\varepsilon^{\cdot *}+\lambda_{3}{\left(\ln\left(\varepsilon^{\cdot *}\right)\right)}^{2}\right)\left(T-T_{r}\right)\right]$$

where $A,\;B_{1},\;B_{2},\;C_{1},\;\lambda_{1},\lambda_{2},$ and $\lambda_{3}$ are material constants. Constants $A,\;B_{1},\;B_{2}$, and $C_{1}$ are determined in the same way that was explained in Section 3.2.5. Taking the logarithm after performing some rearrangements, Equation (77) can be introduced as

(78) $$\ln\left[\frac{\sigma }{\left(A+B_{1}\varepsilon +B_{2}\varepsilon^{2}\right)\left(1+C_{1}\ln\varepsilon^{\cdot *}\right)}\right]=\left(\lambda_{1}+\lambda_{2}\ln\varepsilon^{\cdot *}+\lambda_{3}{\left(\ln\left(\varepsilon^{\cdot *}\right)\right)}^{2}\right)\left(T-T_{r}\right)$$

**Figure 48:**
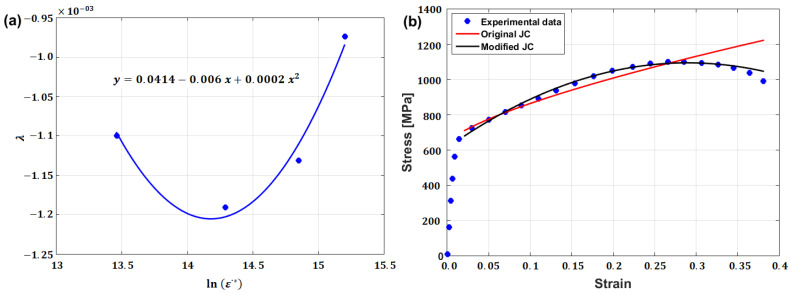
(**a**) Quadratic fitting of parameter λ with $ln\;\left(\varepsilon^{\cdot *}\right)$ to obtain constants $\lambda_{1},\lambda_{2},$ and $\lambda_{3}$ and (**b**) comparison between experimental stresses (markers) and predicted stresses (dashed lines) for TA23 titanium alloy obtained by the original JC model and the modified JC model that was introduced by Yu et al. [60].

By introducing a new parameter, $\lambda =\lambda_{1}+\lambda_{2}\ln\varepsilon^{\cdot *}+\lambda_{3}{\left(\ln\left(\varepsilon^{\cdot *}\right)\right)}^{2}$, different values of λ (the slope of Equation (78)) can be determined at different strain rate and temperature values, which can be fitted as quadratic functions in $\ln\varepsilon^{\cdot *}$; then, constants $\lambda_{1},\lambda_{2},$ and $\lambda_{3}$ can be determined from the fitting (cf. Figure 48a).

Compared with the original JC model, the modified JC model that was presented by Yu et al. [60] prediction a good prediction of the flow behavior of the TA23 titanium alloy under hot deformation, with an RE value of 3.28% compared with 5.32% for original JC model (cf. Figure 48b). The interrelation between the strain rate and temperature, which has been taken into account, might be a reason for the obtained agreement.

#### 3.2.31. Wang (4) JC-Based Modification

**Figure 49:**
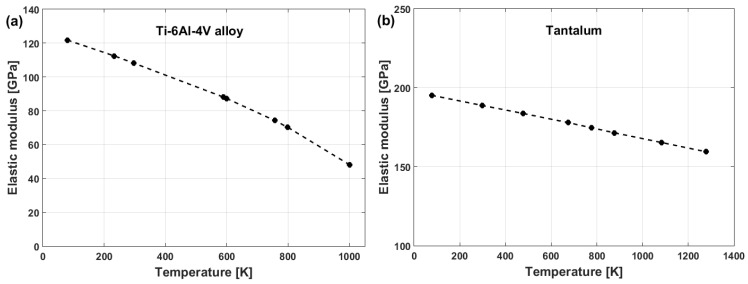
Elastic modulus vs. temperature for (**a**) Ti-6Al-4V alloy and (**b**) tantalum.

Wang et al. [95] modified the softening term in the original JC model to predict the flow behavior of metallic materials at a wide range of strain rates and temperatures. The dependency of temperature on the elastic modulus and Poisson’s ratio is taken into consideration in the modified JC model. The modified JC model that was introduced by Wang et al. [95] can be expressed as

(79) $$\sigma =\left(A+B\varepsilon^{n}\right)\left(1+C\ln\left(\varepsilon^{\cdot *}\right)\right){\left[\frac{\left(1+\mu_{T_{0}}\right)}{\left(1+\mu_{T}\right)}\times \frac{E_{T}}{E_{T_{0}}}\times \frac{T_{m}-T}{T_{m}-T_{0}}\right]}^{0.5}$$

where $T_{0}$ is defined as room temperature; parameters $E_{T}$ and $E_{0}$ are the elastic modulus at the tested temperature and room temperature, respectively; and $\mu_{T}$ and $\mu_{T_{0}}$ are Poisson’s ratio at the tested temperature and room temperature, respectively. Plots of the elastic modulus vs. temperature for the Ti–6Al–4V and tantalum alloys are shown in Figure 49a and Figure 49b, respectively. The modification contains only four constants, A,B,n, and C; however, they must be determined at the same time. Otherwise, regression analysis will not help. Wang et al. [95] presented a multi-objective technique along with the Latin hypercube sampling method, Spearman rank correlation analysis, and a modern genetic algorithm to determine the four constants at once.

**Figure 50:**
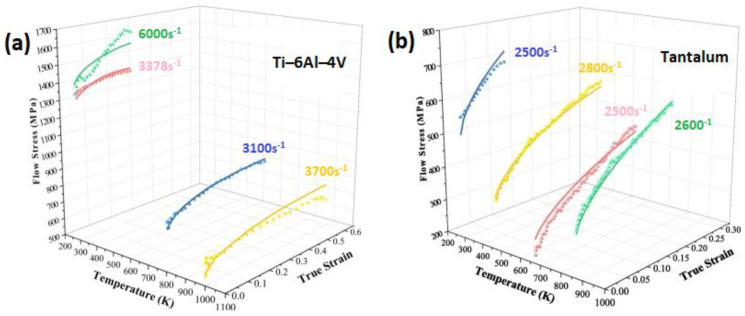
Experimental stresses (markers) compared to predicted stresses (solid lines) for the prediction of flow behavior using the modified JC that was presented by Wang et al. [95] for (**a**) Ti-6Al-4V and (**b**) tantalum.

Comparisons between the experimental stresses and predicted stresses obtained by the modified JC model that was introduced by Wang et al. [95] for the prediction of the flow behavior of Ti–6Al–4V and tantalum are shown in Figure 50a and Figure 50b, respectively. As can be seen, the modified model fits the experimental data for Ti-6Al-4V and tantalum very well.

#### 3.2.32. Shokry (2) JC-Based Modification

Shokry et al. [96] introduced an improved generic modification of the original JC model for flow behavior predictions for different element-based alloys at elevated temperatures and a wide range of strain rates. The coupling effect between the strain and strain rate, as well as between the strain, strain rate, and temperature, is taken into consideration in this modification. The improved generic modification of the original JC model that was introduced by Shokry et al. [96] can be written as

(80) $$\sigma =\left(\overset{3}{\underset{i=0}{\sum }}A_{i}\varepsilon^{i}\right)\left(1+\left(\overset{2}{\underset{i=0}{\sum }}\overset{2}{\underset{j=0}{\sum }}C_{ij\;}\varepsilon^{i}\varepsilon^{\cdot j}\right)\ln\varepsilon^{\cdot *}\right)\exp\left[\left(\overset{2}{\underset{i=0}{\sum }}\overset{2}{\underset{j=0}{\sum }}\overset{2}{\underset{k=0}{\sum }}m_{ijk}\;\varepsilon^{i}\varepsilon^{\cdot j}T^{*k}\right)T^{*}\right]$$

The four constants of $A_{i}$ constitute the strain-hardening term, and the nine constants of $C_{ij\;}$ constitute the strain rate term, while the twenty-seventh constant of $m_{ijk}$ constitutes the softening term. All forty constants are determined using regression analysis in MATLAB. At the reference strain rate and reference temperature, Equation (80) reduces to

(81) $$\sigma =\left(\overset{3}{\underset{i=0}{\sum }}A_{i}\varepsilon^{i}\right)$$

After expansion, Equation (81) extends to four terms with four constants that are constituted with strain and are determined by utilizing regression analysis. At the reference temperature, after performing some rearrangements, Equation (80) is simplified to

(82) $$\left(\frac{\sigma }{\sum_{i=0}^{3}A_{i}\varepsilon^{i}}-1\right)/\ln\varepsilon^{\cdot *}=\overset{2}{\underset{i=0}{\sum }}\overset{2}{\underset{j=0}{\sum }}C_{ij\;}\varepsilon^{i}\varepsilon^{\cdot j}$$

Nine constants are constituted with the strain and strain rate and are determined utilizing regression analysis. At different strain rate values, after performing some rearrangements, Equation (80) is expressed as

(83) $$\frac{\ln\left[\frac{\sigma }{\left(\sum_{i=0}^{3}A_{i}\varepsilon^{i}\right)\left(1+\left(\sum_{i=0}^{2}\sum_{j=0}^{2}C_{ij\;}\varepsilon^{i}\varepsilon^{\cdot j}\right)\ln\varepsilon^{\cdot *}\right)}\right]}{T^{*}}=\overset{2}{\underset{i=0}{\sum }}\overset{2}{\underset{j=0}{\sum }}\overset{2}{\underset{k=0}{\sum }}m_{ijk}\;\varepsilon^{i}\varepsilon^{\cdot j}T^{*k}$$

The right term provides 27 constants of $m_{ijk}$ after an expansion that is constituted by strain, strain rate, and temperature and is determined using regression analysis.

**Figure 51:**
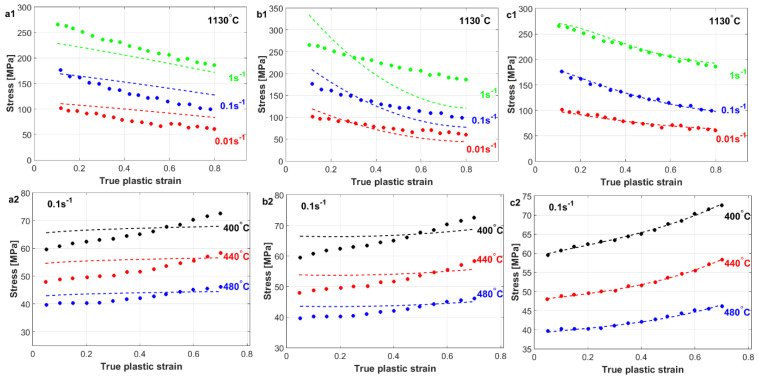
Comparison between experimental stresses (markers) and predicted stresses (solid lines) for U720LI (first raw) and AA7020 (second raw) alloys obtained using (**a1**,**a2**) the original JC model, (**b1**,**b2**) the modified JC model that was presented by Lin et al. [73], and (**c1**,**c2**) the improved generic modified JC model that was presented by Shokry et al. [96].

Precise predictions of the flow behavior of nickel-based (U720LI) and aluminum-based (AA7020) alloys using the improved generic modification of the original JC that was presented by Shokry et al. [96] are achieved (cf. Figure 51c1,c2) compared with experimental stresses, as well as predicted stresses obtained using both original JC model (cf. Figure 51a1,a2) and the modified JC model that was presented by Lin et al. [73] (cf. Figure 51b1,b2). Among the employed models, the improved generic modification provides the highest R, with values of 0.994 ± 0.013, and the lowest AARE, with values of 1.95 ± 1.08%, for the six tested alloys. The very accurate predictions of the flow behavior are due to the coupling effect between the strain, strain rate, and temperature.

#### 3.2.33. Priest JC-Based Modification

Priest et al. [97] introduced a modification of the original JC model for the prediction of machining simulations in C45 steel at different temperatures and different strain rates. Both strain rate and softening terms are modified in the presented modification to take the coupling effect between the temperature and strain rate into account. The modified JC that was introduced by Priest et al. [97] can be written as

(84) $$\sigma =\left(A+B\varepsilon^{n}\right)\left(1+\left(a_{c}\exp\left(b_{c}T\right)+c_{c}\right)\ln\left(\varepsilon^{\cdot *}\right)\right)\left(1-T^{*\left(a_{m}\exp\left(-{\left(\frac{T-b_{m}}{c_{m}}\right)}^{2}\right)\right)}\right)$$

where $A,\;B,n,\;a_{c},\;b_{c},c_{c},a_{m},\;b_{m},$ and $c_{m}$ are material constants. Constants A, B, and n are determined in the same way that was explained with the original JC model (see Section 3.1). At the reference temperature, after performing some rearrangements, Equation (84) can be written in the form of

(85) $$\frac{\sigma }{\left(A+B\varepsilon^{n}\right)}-1=\left(a_{c}\exp\left(b_{c}T\right)+c_{c}\right)\ln\left(\varepsilon^{\cdot *}\right)$$

**Figure 52:**
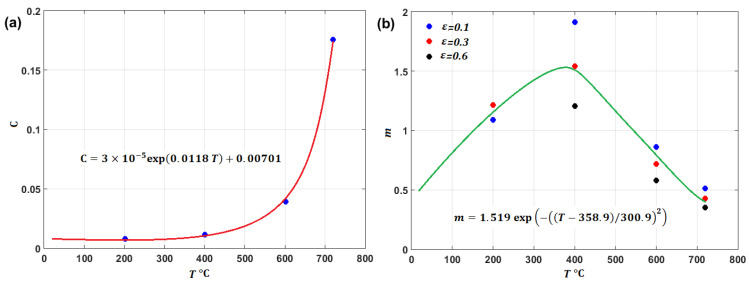
(**a**) Exponential fitting of strain rate parameter vs. temperature; (**b**) gaussian fitting of softening parameter vs. temperature.

Using linear regression, at different temperature and strain rate values, different values for the slope of Equation (85), $a_{c}\exp\left(b_{c}T\right)+c_{c}$, are obtained, which can be exponentially fitted with temperature to obtain constants $a_{c},\;b_{c}$, and $c_{c}$ (cf. Figure 52a). At the reference strain rate, and by taking logarithm after performing some rearrangements, Equation (84) can be written as

(86) $$\ln\left[1-\frac{\sigma }{\left(A+B\varepsilon^{n}\right)}\right]=a_{m}\exp\left(-{\left(\frac{T-b_{m}}{c_{m}}\right)}^{2}\right)\ln T^{*}$$

At different temperatures, different values for the slope of Equation (86), $a_{m}\exp\left(-{\left(\left(T-b_{m}\right)/c_{m}\right)}^{2}\right)$, are obtained, which can be fitted using a gaussian fitting with temperature to obtain constants $a_{m},\;b_{m}$, and $c_{m}$ (cf. Figure 52b).

**Figure 53:**
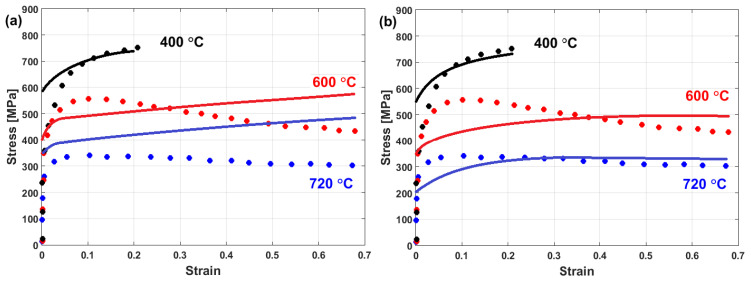
Comparison between experimental stresses (markers) and predicted stresses (solid lines) for C45 steel alloy obtained by (**a**) the original JC model and (**b**) the modified JC model that was presented by Priest et al. [97].

A comparison between the experimental stresses and predicted stresses for the flow behavior of C45 steel alloy under hot deformations obtained using both the original JC model and the modified JC model that was introduced by Priest et al. [97] is shown in Figure 53. The modified model (cf. Figure 53b) provided better predictions than those obtained using the original JC model (cf. Figure 53a).

## 4. Discussions and Summary

The flow behavior of metals and alloys is highly affected by hot working conditions, strain, strain rate, and temperature. As temperature decreases and strain rate increases, strain hardening increases. This is due to the emergence of crystal defects, mainly dislocations, strain-induced stages, or twin boundaries through plastic deformation [190,191,192]. Both the strain strength coefficient and strain-hardening index rise very quickly in the initial strain, followed by a decrease and steady value at high strain rates [14,193]. In general, an increasing strain rate is followed by an increase in flow stress; this is due to the restricted time that is required for dynamic recovery and growth associated with the nucleation of dynamic recrystallization. In contrast, plenty of time leads to a slow rate of dynamic recovery as well as dynamic recrystallization at low strain rates and high temperatures [84,134].

Modeling the flow behavior of metals and alloys at a wide range of temperatures and strain rates is essential to optimize hot working conditions as well as predict the mechanical behavior of metals and alloys with applications under severe conditions such as dynamic loadings and elevated temperatures. The Johnson–Cook model [47] is one of the most important phenomenological models and is widely used to predict the flow behavior of materials at different strain rates and different temperatures. The model contains three independent terms: (i) strain hardening, (ii) strain rate, and (iii) thermal softening. One of the most powerful sides of the JC model is that it has only five constants, which constitute the relationship between flow stress and strain. However, a lot of criticism has been reported vs. the accuracy of predictions of flow behavior using the JC model, particularly with complex non-linear flow stress behavior. As a matter of fact, the JC model does not take the coupling effect of the strain, strain rate, and temperature into consideration in the variation of material parameters, which might be an acceptable reason for the inaccurate predictions of the JC model. Consequently, many modifications of the JC model have been introduced to precisely predict the flow behavior of metals and alloys.

The JC model starts by predicting flow stress using the strain-hardening term, followed by multiplying strain rate and softening terms. The JC model employs the Ludwik equation [194] to represent the strain-hardening term, $\sigma =A+B\varepsilon^{n}$, in which A is yield stress, and B and n are the strain-hardening strength and strain-hardening exponent, in which the strain-hardening exponent considers the balance between softening and work hardening. The JC model achieves good agreement for strain hardening between the experimental and predicted stresses using the Ludwik equation for different alloys, such as nickel [48], iron [30], aluminum [51,52], and titanium-based [60] alloys. In order to describe the saturation of strain hardening, as well as the continuance of strain hardening with the progress in strain, Shin and Kim [75], replaced the Ludwik equation with the Voce hardening [180] equation in a modified JC model, which is represented by $\sigma =\left[A+B\left\{1-\exp\left(-C\varepsilon \right)\right\}\right]$, in which A represents yield stress, constants B and C introduce saturated strain hardening and the strain-hardening index respectively. Good agreements between the experimental and predicted stresses for copper can be obtained. Similar findings were obtained using Voce hardening as a modification term in the original JC model that was presented by Wang et al. [77] for 30Cr2Ni4MoV rotor steel alloy. To overcome the difficulty of finding the real value of the yield stress, the yield stress constant, A, is replaced by beak stress, $\sigma_{0}$, using the Zener–Hollomon parameter, which is provided by $\sigma_{0}=\left(1/\alpha \right)\ln\left\{{\left(Z/A\right)}^{1/m}+{\left[{\left(Z/A\right)}^{2/m}+1\right]}^{1/2}\right\}$, where Z, α, A, and m are defined in Section 3.2.10. This modification of the strain-hardening term is introduced with good predictions of the flow stress of Al-Zn-Mg-Cu alloy [78] and 7075 Al alloy [79]. A different replacement for the strain-hardening term of the original JC model was presented by Lin et al. [73], in which the stress–strain data at both the reference strain rate and reference temperature are fitted using quadratic function as $A+B_{1}\varepsilon +B_{2}\varepsilon^{2}$ with material constants $A,B_{1}$, and $B_{2}$. A similar replacement with a quadratic polynomial was implemented as in [60,81] for TA23 titanium alloy and titanium matrix composite, and with polynomial fitting using a three-order function as in [84,85,96] for alloy 800H, FeCr alloy, and different element-based alloys.

The strain rate term has received a lot of modifications in the original JC model, which can be implemented when studying hot deformation as well as dynamic loadings. Rule and Jones [68] modified the strain rate term in the original JC model to mimic the quick rise in stress due to dynamic loadings, with good predictions of flow stress for different alloys such as 7075-T6 aluminum and high-strength steel alloys, in which the term $C_{4}\left(1/\left(C_{5}-\ln\varepsilon^{\cdot *}\right)-1/C_{5}\right)$ is added to the strain rate term in the original JC model, where $C_{4}$ and $C_{5}$ are material constants. Another modification of the prediction of the dynamic behavior of a vehicle body under a very high strain rate was presented by Kang et al. [69], in which the strain rate term is expressed as a quadratic function of the logarithm of the strain rate by adding the term $C_{2}{\left(\ln\varepsilon^{\cdot *}\right)}^{2}$ to the strain rate term in the original JC model, and $C_{2}$ is a material constant, which gave provided predictions for steel plate material. Good predictions can also be obtained when using the same strain rate modification for Ti-6Al-4V tubes [63] and ballistic Roma Plastilina No. 1 clay [92]. Another modification was introduced by considering parameter C in the strain rate term of the original JC model depending on the strain, with a linear relationship [77,84] for the predictions of the flow behavior of 30Cr2Ni4MoV rotor steel and alloy 800H and a quadratic relationship [86] to predict the flow behavior of 10% Cr steel alloy. More interpretations of the strain rate term as quadratic functions in both the strain and strain rate are presented in [90,93,94,96], with good agreements between the experimental and predicted stresses. Couque et al. [71] obtained good predictions of the flow behavior of nickel when an exponential term is added to the strain rate in the original JC model as $E{\left(\varepsilon^{\cdot }/\varepsilon^{\cdot }{}_{1}\right)}^{k}$, in which E and k are material constants. A sine wave representation of the strain rate term is introduced to modify the original JC model in [48,85,88] for the prediction of the flow behavior of Inconel 718, FeCr, and SnSbCu alloys, accompanied by good predictions.

The thermal-softening term in the original JC model has also received a lot of modifications. The early modified softening term was introduced by Meyers et al. [67], in which the softening term in the original JC model is replaced by an exponential term $e^{-\lambda \left(T-T_{r}\right)}$, and λ is a material constant. Lin et al. [73] introduced a modification of the softening term that takes the coupling effect between the strain rate and temperature into account, considering the parameter $\lambda =\lambda_{1}+\lambda_{2}\ln\varepsilon^{\cdot *}$, in which $\lambda_{1}$ and $\lambda_{2}$ are two constants that constitute the linear relationship between λ and $\ln\varepsilon^{\cdot *}$. This modification has been widely used, with good predictions of flow behavior for different alloys, such as 28CrMnMoV steel [170], Hastelloy C-276 alloy [171], 40CrNi steel [172], and 2297 Al-Li alloy [174]. It is also employed in other modifications of the original JC model, as in [77], with 30Cr2Ni4MoV rotor steel alloy and [86] 10%Cr steel alloy. The same modification, but with a quadratic relationship instead of a linear one, provided $\lambda =\lambda_{1}+\lambda_{2}\ln\varepsilon^{\cdot *}+\lambda_{3}{\left(\ln\left(\varepsilon^{\cdot *}\right)\right)}^{2}$ for the prediction of the flow behavior of TA23 titanium alloy, which was utilized in [60]. Another modification of the softening term was introduced by Hou Q. Y. et al. [74] to overcome the problem of predicting flow behavior at a lower temperature than the reference temperature. In this modification, $T^{*}$ in the original JC model is replaced by $\lambda \left[\left(e^{T/T_{m}}-e^{T_{r}/T_{m}}\right)/\left(e-e^{T_{r}/T_{m}}\right)\right]$, which provides good predictions for Mg–10Gd–2Y–0.5Zr alloy. The parameter λ is replaced by λ′ε in another modification that was presented by Perez et al. [178] so that $\lambda^{\prime }$ has a constant value considering the change in strain. Lin et al. [78] replaced constant m in the original JC model with a linear function in the strain rate as $0.71291+0.04391\varepsilon^{\cdot }$, with good agreement with the prediction of the flow behavior of a Zn-Mg-Cu alloy with experimental stresses. In the same way, constant m is replaced by a linear relationship but in the strain as $m_{1+}m_{2}\varepsilon$ for predictions of flow behavior of alloy 800H [84] and CuCrZr alloy [93], providing good predictions. By using the fourth polynomial equation in temperature, the m constant is modified as $a+bT^{*}+cT^{*}{}^{2}+dT^{*}{}^{3}+eT^{*}{}^{4}$ in another modification that was presented by Tao et al. [63] for the prediction of the flow behavior of Ti-6Al-4V, in which constants a,b,c,d, and e are determined using polynomial fitting. The strain rate is coupled with the quadratic function of temperature in another modification for the softening term in the original JC model, which was introduced by Song et al. [81] for the prediction of the flow behavior of a titanium matrix composite and expressed as $\exp\left(\lambda_{1}\;T^{*}+\lambda_{2}T^{*}{}^{2}\;\right)\ln\varepsilon^{\cdot *}$, providing good predictions. Interactions between the effects of the strain, strain rate, and temperature are taken into consideration in other modifications, in which constant λ, which was introduced in [67], is replaced by a third-order multiplier of the strain, strain rate, and temperature, with eight selected terms in [90] for the prediction of A356 alloy and with twenty-seven terms in [96] for the prediction of four different element-based alloys, providing precise predictions.

## 5. Conclusions and Future Directions

In this review article, the flow behavior of metals and alloys at a wide range of strain rates and different temperatures is studied. Thus, the constitutive model of the original JC model, as well as more than thirty modified JC-based models, are critically reviewed and commented on. In addition, the methods and techniques that are used to determine model constants are presented and explained. Finally, a summary of modifications based on the three terms of the original JC model is presented.

The combination of multiple factors and their interactions, which may affect the flow stress response, is a much more efficient research methodology for empirical modeling. The Johnson–Cook model is a strong phenomenological model that has been extensively used for predictions of the flow behavior of metals and alloys. It has been implemented in finite element software packages, which enhances its importance in performing simulation analysis of hot working processes; optimizing strain, strain rate, and temperature; and simulating real applications in severe conditions. The findings of this review can be summarized as follows:

- The Johnson–Cook model has been widely used to predict the flow behavior of metals and alloys at a wide range of temperatures and strain rates. In this regard, modified JC-based models were introduced for accurate predictions of the flow behavior for metals and alloys with complex non-linear behavior in which the JC model fails to precisely predict the flow behavior.
- Lin et al. [73] provided one of the most important modifications of the original JC model, which has been used for the prediction of the flow behavior of different metals and alloys. The modification provides good predictability of flow behavior when compared with experimental stresses in many different alloys.
- The improved generic modification for the JC model that was introduced by Shokry et al. [96] can be considered one of the most promising modifications, in which the interaction between the strain, strain rate, and temperature are taken into account. Accurate predictions are obtained using the improved generic JC model when implemented in six different element-based alloys. One of the limitations of this modification is the large number of constants it has: forty constants. However, this problem is not a concern nowadays due to the rapid improvements in computers and the software used for the determination of the constants.
- Comparing predicted stresses using the original JC model and the modified JC-based models for the same types of alloys might be a future direction to precisely assess and evaluate the predictability of the JC model and modified JC-based models.
- Another future direction is considering the inverse analysis methods and the techniques that are based on non-linear least squares methods to minimize the mean square error between experimental and predicted values for the accurate determination of model constants.
- Coupling the original JC model and the modified JC-based models with other models such as the Zerilli–Armstrong and Arrhenius models might be another future direction.

## Figures and Tables

**Table 1:** Values of computed $V\left(\varepsilon^{\cdot },\varepsilon \right)$ at different strain rates (×10^−4^).

Strain Rate	Strain								
	0.1	0.15	0.2	0.25	0.3	0.35	0.4	0.45	0.5
0.01 s^−1^	0.266	0.196	0.825	1.10	1.59	1.60	2.42	2.35	2.6
0.1 s^−1^	2.14	1.77	1.85	1.99	2.17	2.31	2.57	2.79	3.32
1 s^−1^	−0.593	−0.54	−0.61	−0.49	−0.26	−0.026	0.269	0.665	0.988

## Data Availability

Data sharing not applicable.
